# Exofacial membrane composition and lipid metabolism regulates plasma membrane P4-ATPase substrate specificity

**DOI:** 10.1074/jbc.RA120.014794

**Published:** 2021-01-13

**Authors:** Bhawik Kumar Jain, Bartholomew P. Roland, Todd R. Graham

**Affiliations:** Department of Biological Sciences, Vanderbilt University, Nashville, Tennessee, USA

**Keywords:** P4-ATPase, membrane biology, membrane asymmetry, flippase, sphingolipids, glycosphingolipids, lipid transport, sphingolipid, membrane transport, plasma membrane, glycolipid

## Abstract

The plasma membrane of a cell is characterized by an asymmetric distribution of lipid species across the exofacial and cytofacial aspects of the bilayer. Regulation of membrane asymmetry is a fundamental characteristic of membrane biology and is crucial for signal transduction, vesicle transport, and cell division. The type IV family of P-ATPases, or P4-ATPases, establishes membrane asymmetry by selection and transfer of a subset of membrane lipids from the lumenal or exofacial leaflet to the cytofacial aspect of the bilayer. It is unclear how P4-ATPases sort through the spectrum of membrane lipids to identify their desired substrate(s) and how the membrane environment modulates this activity. Therefore, we tested how the yeast plasma membrane P4-ATPase, Dnf2, responds to changes in membrane composition induced by perturbation of endogenous lipid biosynthetic pathways or exogenous application of lipid. The primary substrates of Dnf2 are glucosylceramide (GlcCer) and phosphatidylcholine (PC, or their lyso-lipid derivatives), and we find that these substrates compete with each other for transport. Acutely inhibiting sphingolipid synthesis using myriocin attenuates transport of exogenously applied GlcCer without perturbing PC transport. Deletion of genes controlling later steps of glycosphingolipid production also perturb GlcCer transport to a greater extent than PC transport. In contrast, perturbation of ergosterol biosynthesis reduces PC and GlcCer transport equivalently. Surprisingly, application of lipids that are poor transport substrates differentially affects PC and GlcCer transport by Dnf2, thus altering substrate preference. Our data indicate that Dnf2 exhibits exquisite sensitivity to the membrane composition, thus providing feedback onto the function of the P4-ATPases.

The plasma membrane is organized as a lipid bilayer consisting of exofacial and cytofacial leaflets. Different lipids are distributed heterogeneously between these two leaflets to form an asymmetric membrane structure. The exofacial side of the mammalian plasma membrane is enriched with phosphatidylcholine (PC) and sphingolipids (sphingomyelin and glycosphingolipids), and the cytosolic side is enriched for phosphatidylserine (PS) and phosphatidylethanolamine (PE) (1, 2, 3). This asymmetric nature of the lipid bilayer is implicated in different cellular processes, such as vesicular trafficking, apoptosis, signal transduction, and cytokinesis. The alteration of lipid asymmetry also plays an important role in physiological functions like blood coagulation and host–pathogen interactions (4).

Membrane asymmetry is generated primarily by ATP-dependent lipid transporters called P4-ATPases, or flippases. These integral membrane enzymes transport lipid substrates from the exofacial to the cytosolic leaflets of the membrane. P4-ATPases are heterodimers with a catalytic α subunit (P4-ATPase) and a noncatalytic β subunit (Cdc50, Lem3, or Crf1 in yeast; CDC50A, CDC50B, or CDC50C in humans). The budding yeast *Saccharomyces cerevisiae* expresses 5 P4-ATPases: Dnf1, Dnf2, Dnf3, Drs2, and Neo1, whereas 14 different P4-ATPases are expressed in humans (5, 6, 7). Mutations in these human P4-ATPases are associated with neurological disease, cholestasis, reproductive dysfunction, and metabolic disease (8, 9)

Understanding the substrate specificity of these transporters is essential for determining their role in health and disease. P4-ATPases were first described to be aminophospholipid (PS and PE) translocases and have traditionally been thought to specifically transport glycerophospholipids from the exofacial to the cytofacial side of the bilayer (10). Recently, however, we and others discovered that a group of yeast and human P4-ATPases translocate the sphingolipid glucosylceramide (GlcCer). GlcCer is the primary substrate for the human P4-ATPases ATP10B and ATP10D, whereas ATP10A transports both PC and GlcCer (11, 12). GlcCer is a central intermediate in sphingolipid biosynthesis and acts as a precursor for the synthesis of all complex glycosphingolipids, such as gangliosides and globosides in animal cells (13). GlcCer accumulation has been associated with Gaucher and Parkinson's diseases. Indeed, mutations in the endo/lysosomal ATP10B are also linked to Parkinson's disease, cause accumulation of GlcCer and lysosomal dysfunction, and loss of cortical neurons (12). In addition, mutations in ATP10A and ATP10D are associated with diet-induced obesity, insulin resistance, myocardial infarction, and atherosclerosis (14, 15, 16, 17, 18, 19). Thus, GlcCer metabolism and transport play crucial roles in various pathologies.

The GlcCer flippases in yeast are Dnf1 and Dnf2, orthologs of the ATP10A/B/D group in mammals (11). Dnf2 is the primary plasma membrane GlcCer flippase in *S. cerevisiae*, and this protein transports the secondary and tertiary substrates PC and PE. Dnf1 also transports GlcCer, PC, and PE but mostly localizes to intracellular compartments of the cell (10, 11, 20). A conserved Gln residue located in the middle of transmembrane segment 4 is critical for GlcCer transport, and Asn substitutions are sufficient to ablate GlcCer translocation in human and yeast enzymes without substantially altering recognition of glycerophospholipid substrates (11). Dnf1 and Dnf2 have been shown to be involved in sphingolipid homeostasis through their regulation by the flippase protein kinases (Fpk1/2) (21). Dnf1 and Dnf2 require phosphorylation by Fpk1/2 in the consensus motif R*X*Sϕ(D/E) for activity (22, 23). Fpk1/2 supports metabolic responses to changes in sphingolipid homeostasis through their connection to the TORC2 signaling network with yeast orthologs of PKD1 (Pkh1) and SGK1 (Ypk1). Most fungi produce both GlcCer and inositol phosphorylceramide (IPC), but *S. cerevisiae* lacks GlcCer synthase and does not synthesize this lipid (24). IPC can be further modified with a second inositol phosphate group and/or mannose within the lumen of the Golgi to form MIPC and M(IP)_2_C, lipids that occupy the plasma membrane extracellular leaflet (25, 26, 27).

The plasma membrane has a tremendous diversity of lipid species, and P4-ATPases must sort through these lipids to establish membrane asymmetry. The identification and selection of which lipids to translocate and which to leave behind could be modulated by the composition of the membrane environment. The exofacial membrane leaflet consists of various lipid molecules that could be either high-affinity or low-affinity substrates, potential inhibitors of transport, or allosteric modulators of the P4-ATPases. In our previous studies, we showed that unlabeled glucosylsphingosine (GlcSph) and lyso-PC, lipids lacking a fatty acyl chain, could inhibit NBD-GlcCer transport by Dnf2 (11). These results suggested that the two substrate lipids are competing for a common transport pathway in the P4-ATPase or that changing the membrane composition influences flippase activity. In this study, we find that acute inhibition of sphingolipid synthesis or application of exogenous lipids modulates flippase substrate specificity.

## Results

### Acute inhibition of sphingolipid synthesis alters the substrate preference of Dnf1 and Dnf2

**Figure 1:**
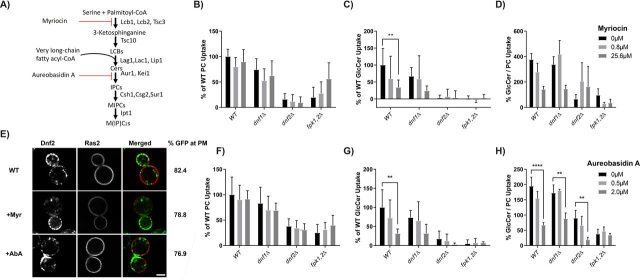
**Acute inhibition of sphingolipid synthesis selectively reduces NBD-GlcCer transport.** (*A*) Sphingolipid biosynthesis pathway in *S. cerevisiae* leading to the synthesis of IPC, MIPC and M(IP)_2_C including steps inhibited by myriocin and aureobasidin A. Yeast strains BY4741 (WT), *dnf1*Δ, and *dnf2*Δ strains were grown to the mid-log phase and treated with different concentrations of myriocin (*B*–*D*) or aureobasidin A (*F*–*H*) for 30 min at room temperature. Inhibitor treated cells were incubated with NBD-PC (*B*, *F*) or NBD-GlcCer (*C*, *G*) for 30 mins and lipid uptake was measured using flow cytometry. Lipid uptake activities were plotted as the percentage of NBD-PC or NBD-GlcCer uptake for mock treated (0 μm) wild-type cells. (*D*, *H*) The GlcCer/PC ratio provides a measure of substrate preference. Statistical variance was tested using one-way ANOVAs test and Tukey's post hoc analysis. *, *p* < 0.05; **, *p* < 0.01; ***, *p* < 0.001. *Error bars* ± S.D. (*n* ≥ 9). (*E*) Wild type yeast strain expressing Dnf2-mNG and mCherry-Ras2 were treated with myriocin or aureobasidin A for 1 hr at room temperature and imaged as described under Materials and Methods Representative images are shown. *Scale bar*, 2 μm. Percentage of Dnf2-GFP at the plasma membrane are quantified by drawing circles inside and outside the plasma membrane using Image J to quantify the internal fluorescence and total fluorescence, respectively (*n* = 60).

*S. cerevisiae* is part of a small clade of fungi that have evolutionarily lost the ability to make GlcCer and yet have retained GlcCer transporters (11, 24). Thus, Dnf1 and Dnf2 may facilitate uptake of GlcCer from exogenous sources as fungi are heterotrophic eukaryotic organisms that absorb materials, including lipids, from their environment to facilitate growth (28, 29, 30). We hypothesized that inhibition of endogenous sphingolipid synthesis might enhance GlcCer uptake as a compensatory mechanism. Myriocin blocks sphingolipid biosynthesis at the first step in this pathway by inhibiting serine palmitoyl transferase (31), whereas aureobasidin A inhibits the IPC synthase encoded by the *AUR1* gene (32) (Fig. 1*A*). To measure the uptake of lipid substrate, we used PC, PE, and GlcCer analogs bearing 7-nitro-2-1,3-benzoxadiazol-4-yl (NBD) head group (33, 34, 35). WT, *dnf1*Δ, dn*f2*Δ, and *fpk1,2*Δ (flippase protein kinase) strains were treated with myriocin and aureobasidin A for 30 min, and the uptake of NBD-PC and NBD-GlcCer was measured by flow cytometry (Fig. 1). A *dnf1,2*Δ strain was also treated with the NBD lipids, and background (transport-independent) fluorescence was subtracted. The remaining P4-ATPases (Neo1, Drs2, and Dnf3) are primarily localized within intracellular compartments and do not measurably contribute to plasma membrane transport activity for the substrates analyzed (20, 36).

No significant change in uptake of NBD-PC was observed upon treatment with myriocin or aureobasidin A for any of the strains tested (Fig. 1, *B* and *F*). However, NBD-GlcCer uptake was selectively reduced in WT, *dnf1*Δ, and *dnf2*Δ strains treated with myriocin and aureobasidin A (Fig. 1, *C* and *G*). Most of the plasma membrane transport activity is catalyzed by Dnf2 as indicated by the near ablation of transport in the *dnf2*Δ mutant. Interestingly, Dnf1 and Dnf2 retained a weak ability to transport NBD-PC in the *fpk1,2*Δ strain (∼20% of WT transport activity in these experiments) but completely lost the ability to transport NBD-GlcCer. NBD-PC transport trended upward upon inhibition of sphingolipid synthesis in *fpk1,2*Δ, but these differences were not significant. A measure of substrate preference for these flippases is described by the ratio of GlcCer transport to PC transport. The reduced GlcCer/PC uptake ratio in myriocin and aureobasidin A–treated cells indicate that inhibition of sphingolipid biosynthesis specifically affects the GlcCer flippase activity of Dnf2 in a dose-dependent manner (Fig. 1, *D* and *H*). The localization of mNeonGreen (mNG)–tagged Dnf2 to the plasma membrane, marked with Ras2-mCherry, was unaffected by inhibiting sphingolipid synthesis (Fig. 1*E*). Thus, rather than enhancing GlcCer uptake by Dnf1 and Dnf2, inhibition of sphingolipid synthesis alters the substrate preference of these flippases by selectively reducing GlcCer transport.

### Sphingolipid biosynthesis mutants influence the substrate specificity of flippases

**Figure 2:**
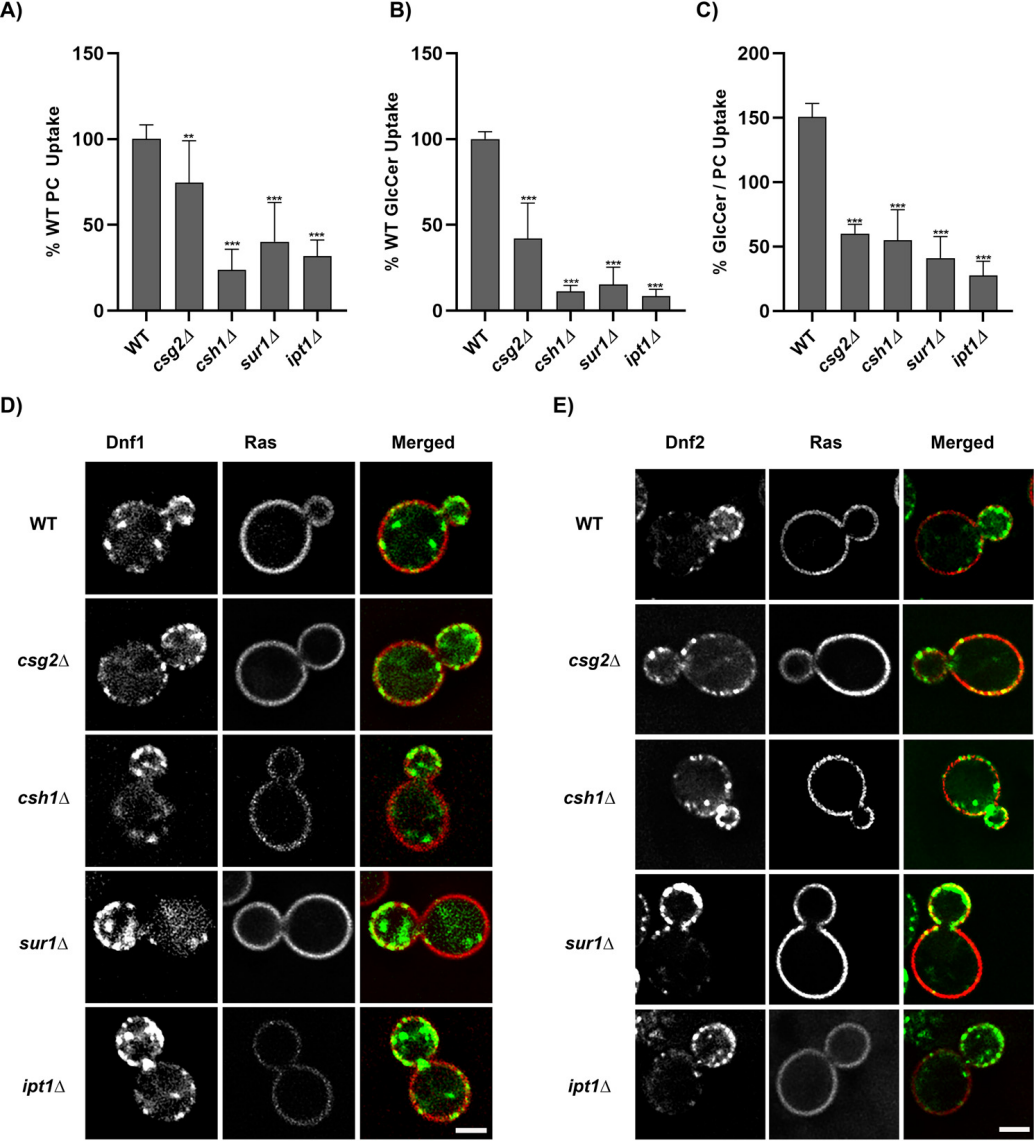
**Perturbations to sphingolipid biosynthesis reduces plasma membrane flippase activity and alters substrate preference.** Yeast strains BY4741 (WT), *dnf1*,2Δ *csg2*Δ, *csh1*Δ, *sur1*Δ, and *ipt1*Δ were grown to mid-log phase and incubated with NBD-PC or NBD-GlcCer for 30 min. *A–C*, lipid uptake activities were plotted as the percentage of NBD-PC (*A*) or NBD-GlcCer (*B*) uptake for WT cells and the GlcCer/PC ratio (*C*). Statistical variance was tested using one-way ANOVA test and Tukey's post hoc analysis. *, *p* < 0.05; **, *p* < 0.01; ***, *p* < 0.001. *Error bars* ± S.D. (*n* ≥ 9). *D*, strains BY4741 (WT), *csg2*Δ, *csh1*Δ, *sur1*Δ, and *ipt1*Δ expressing GFP-tagged Dnf1 and plasma membrane marker Ras2-mCherry were grown to mid-log phase in SD/−Leu, URA medium. The cells were washed and resuspended in minimal medium for imaging. The images were captured using the DeltaVision Elite imaging system (GE Healthcare Life Sciences) equipped with a 100× objective lens followed by deconvolution using softWoRx software. The images were processed using ImageJ. Representative images are shown. *Scale bar*, 2 μm. *E*, yeast strains expressing mNG-tagged Dnf2 and Ras2-mCherry were grown to mid-log phase in SD/−Leu medium. The cells were prepared, measured, and analyzed as outlined in *D*.

The major sphingolipids in *S. cerevisiae* (IPC, MIPC, and M(IP)_2_C) are synthesized from ceramide by Aur1, Csg2, Csh1, Sur1, and Ipt1 (Fig. 1*A*) (37, 38, 39). We tested whether knocking out nonessential biosynthetic genes required to produce mature glycosphingolipids would alter substrate specificity. All sphingolipid biosynthetic mutants exhibited a reduced uptake of NBD-PC, NBD-PE, and NBD-GlcCer (Fig. 2, *A* and *B*, and Fig. S1A). Compared with NBD-PC and NBD-PE, these mutants displayed a greater reduction in flippase activity for NBD-GlcCer and therefore a reduced GlcCer/PC and GlcCer/PE transport ratio (Fig. 2*C* and Fig. S1B). The change in substrate preference could be partly explained by a greater degree of Dnf1 localization to the plasma membrane of the sphingolipid mutants, because this flippase transports NBD-PC and NBD-GlcCer at near equal rates (11). However, we found no change in localization of GFP-Dnf1 or Dnf2-mNG in sphingolipid biosynthesis mutants relative to WT cells (Fig. 2, *D* and *E*, and Fig. S2, A and B). We also observed no change in the polarized localization of Dnf2 by measuring the Dnf2-mNG tip to hip ratio (fluorescence intensity at the bud tip divided by the fluorescence intensity of the mother cell rear) (Fig. S2C). Thus, chronic loss of mature sphingolipids caused a reduction in Dnf1/2 activity and a change in substrate preference toward PC/PE without altering localization of these proteins.

**Figure 3:**
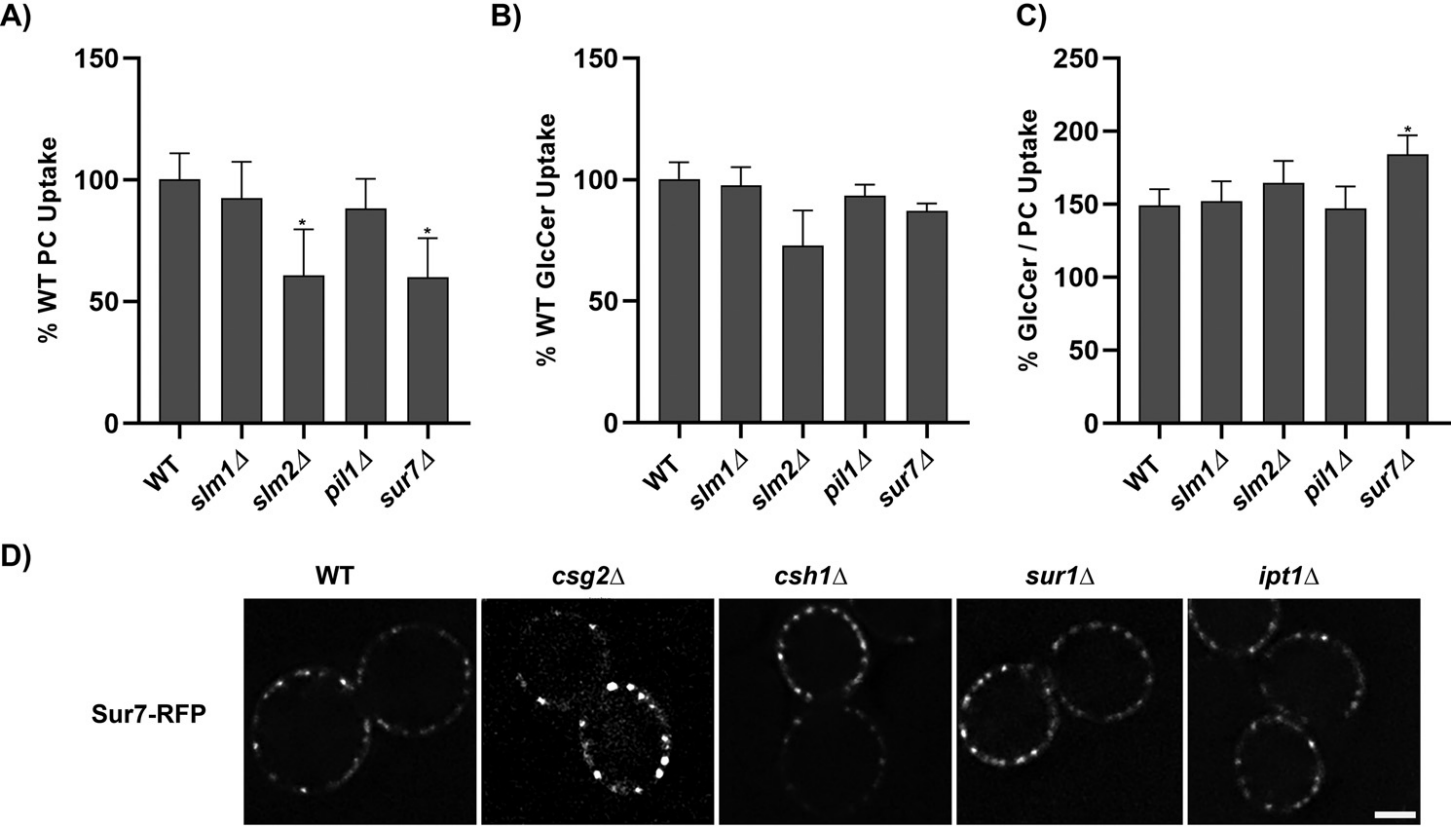
**Influence of eisosome mutants on flippase activity.** Yeast strains BY4741 (WT), *dnf1,2*Δ *slm1*Δ, *slm2*Δ, *pil1*Δ, and *sur7*Δ were grown to mid-log phase and incubated with NBD-PC or NBD-GlcCer for 30 min. *A–C*, lipid uptake activities were plotted as the percentage of NBD-PC (*A*) or NBD-GlcCer (*B*) uptake for WT cells, and the GlcCer/PC ratio (*C*). Statistical variance was tested using one-way ANOVA test and Tukey's post hoc analysis. *, *p* < 0.05. *Error bars* ± S.D. (*n* ≥ 9). *D*, yeast strains BY4741 (WT), *csg2*Δ, *csh1*Δ, *sur1*Δ, *ipt1*Δ, and *fpk1,2*Δ expressing RFP-tagged Sur7 were grown to mid-log phase in SD/−Leu medium. The cells were washed and resuspended in minimal medium for imaging. Images were processed using ImageJ tool. Representative images are shown. *Scale bar*, 2 μm.

Eisosomes are plasma membrane subdomains that play important roles in regulation of plasma membrane organization and have been linked to sphingolipid metabolism, the Pkh/Ypk signaling hub, and Slm1/Slm2 membrane stress sensors (Fig. S3) (40, 41). Therefore, we also tested eisosome structural component mutants (*pil1*Δ and *sur7*Δ) along with *slm1*Δ and *slm2*Δ to determine whether these perturbations affect flippase activity or substrate specificity. We did not observe a significant difference in the uptake of NBD-PC or NBD-GlcCer in *slm1*Δ or *pil1*Δ strains relative to WT, and *slm2*Δ showed a small decrease in both activities. Interestingly, the uptake of NBD-PC was specifically reduced in the *sur7*Δ strain, whereas NBD-GlcCer transport was unchanged (Fig. 3, *A–C*). We further examined eisosome structure in the sphingolipid synthesis mutants but found no difference in Sur7-RFP localization or appearance and eisosome number (Fig. 3*D* and Fig. S2D). Therefore, these observations collectively suggest that eisosomes are not strong mediators of metabolic changes linked to flippase activity, but *sur7*Δ uniquely enhanced substrate preference toward GlcCer.

### Mutation of fpk phosphorylation sites in Dnf2 to alanine ablates flippase activity

**Figure 4:**
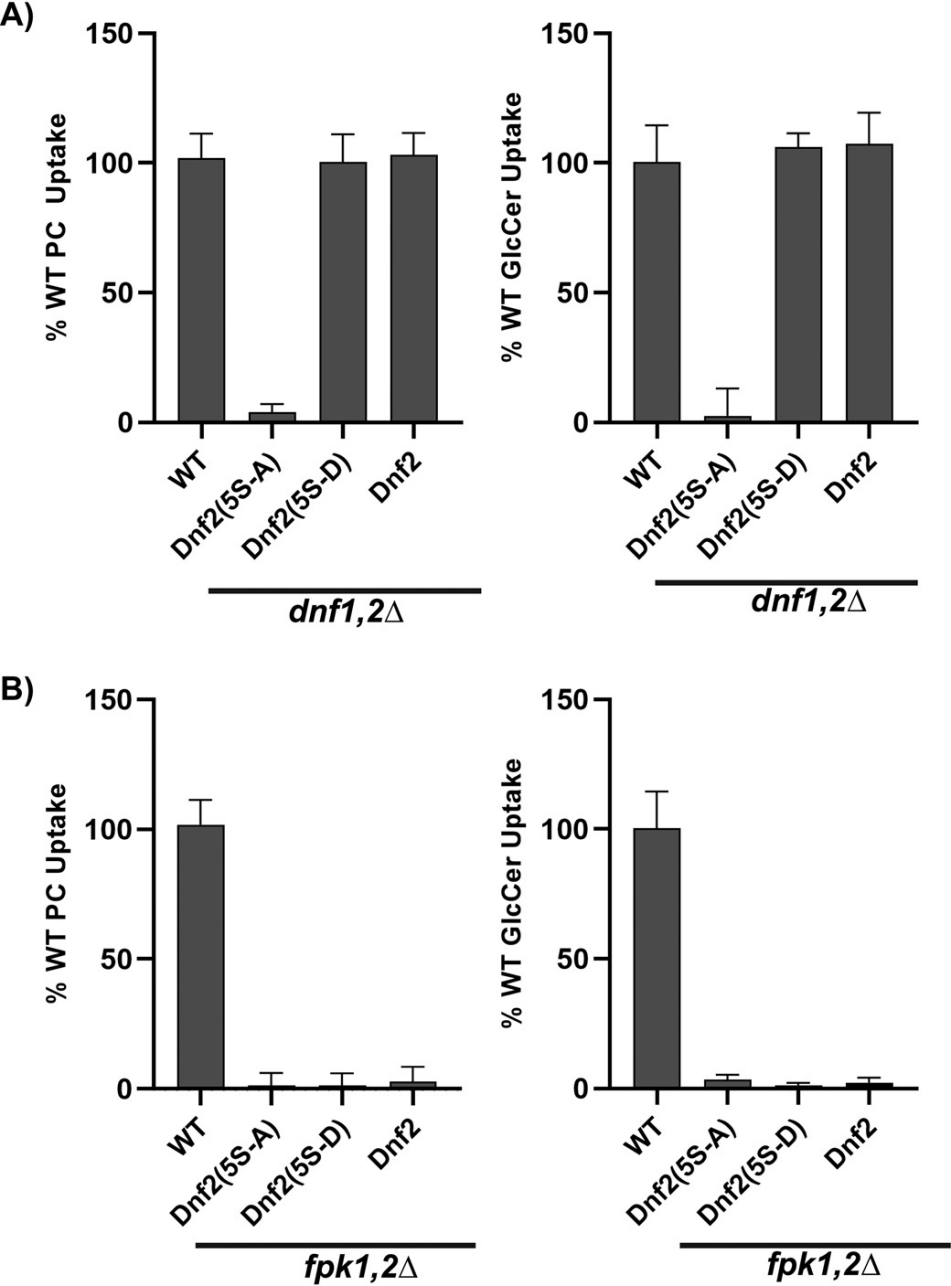
**Fpk phosphorylation sites in Dnf2 are required for both NBD-PC and NBD-GlcCer transport activity.** NBD-PC and NBD-GlcCer uptake was assayed in *dnf1,2*Δ (*A*) and *fpk1,2*Δ (*B*) strains harboring pRS313, pRS313-Dnf2(5S-5A), pRS313-Dnf2(5S-5D), and pRS313-Dnf2 plasmids. Lipid uptake activities were plotted as the percentage of NBD-PC and NBD-GlcCer uptake for WT cells and GlcCer/PC ratio (*n* ≥ 9) ± S.D.

Inhibition of sphingolipid biosynthesis may influence flippase activity by reducing flippase protein kinase (Fpk1 and Fpk2) activity (Fig. S3). Dnf1 and Dnf2 are phosphorylated by Fpk1,2, and their phosphorylation strongly up-regulates flippase activity (21), although the influence on GlcCer transport activity has not been assessed. We hypothesized that Fpk1,2 may be changing Dnf1 and Dnf2 substrate specificity through differential phosphorylation of these flippases (21, 22). Dnf2 possesses five Fpk1,2 phosphorylation sites of the consensus sequence R*X*SLD (Fig. S4) (22). We mutated the serine residues in all five sites (5S-A, 5S-D) to prevent or mimic consensus Fpk1,2 phosphorylation. We sought to produce a gain-of-function *DNF2-5SD* allele to distinguish effects of phosphorylation *versus* more direct effects of membrane composition on Dnf2 activity. The 5S-A mutations resulted in loss of Dnf2 transport activity for both NBD-GlcCer and NBD-PC, without any change in flippase substrate specificity, implying that these residues are critical for flippase activity. Conversely, the Dnf2(5S-D) variant was functional and displayed equivalent transport activity to WT Dnf2 (Fig. 4*A*). However, despite the robust activity of the phosphomimetic Dnf2, this allele could not suppress the lipid transport defects of the *fpk1,2* null strain (Fig. 4*B*). Therefore, *DNF2(5S-D)* does not appear to be a gain-of-function allele capable of bypassing a flippase protein kinase deficiency, and we did not further test this variant in the sphingolipid synthesis mutants.

### Pharmacological and genetic inhibition of ergosterol biosynthesis inhibits flippase activity

**Figure 5:**
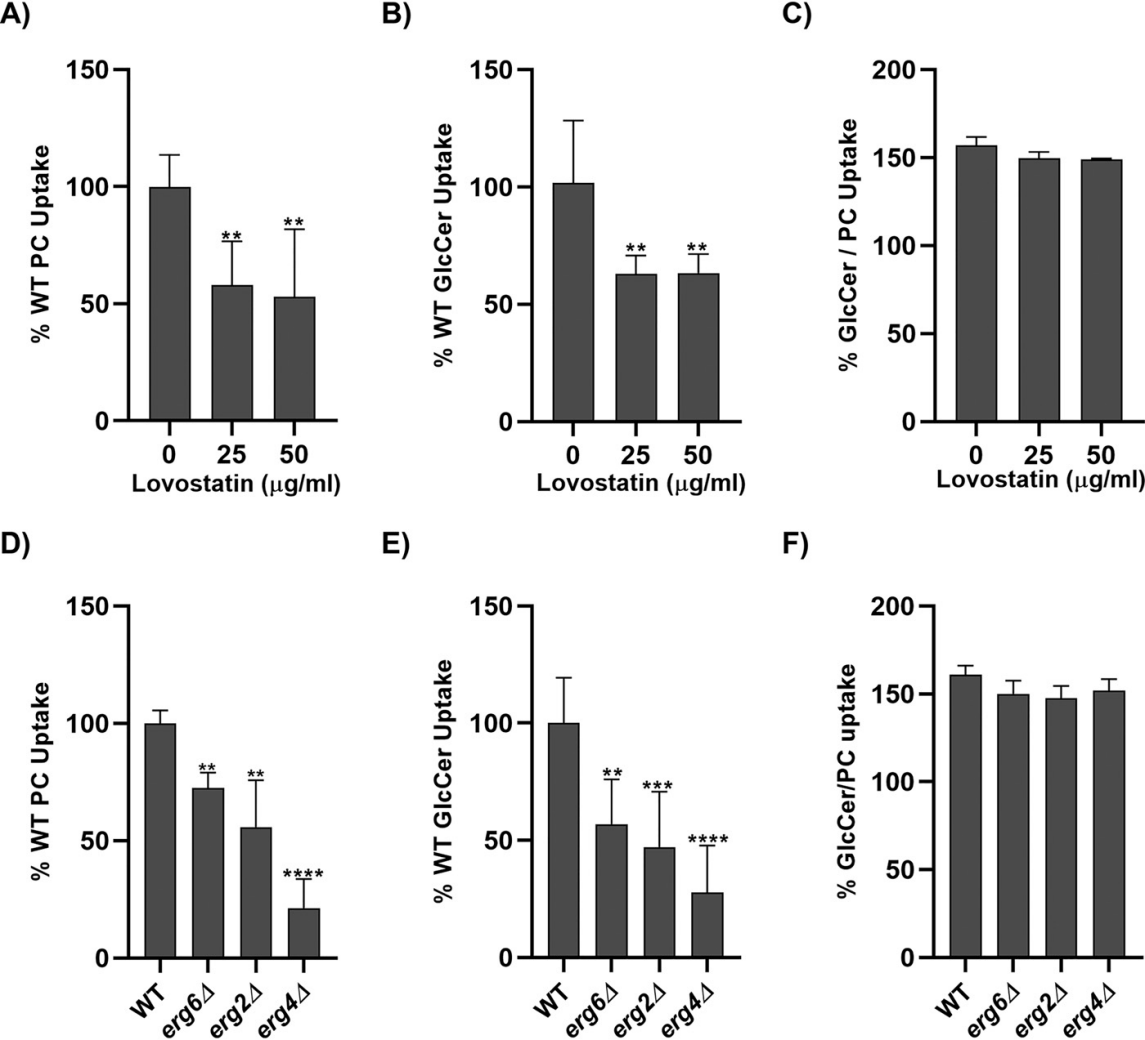
**Acute or chronic perturbation of ergosterol synthesis abrogates Dnf transport activity without altering substrate specificity.** BY4741 (WT) and *dnf1,2*Δ strains were grown to mid-log phase and treated with different concentrations of lovastatin for 1 h at 30° C. Lovastatin-treated cells were incubated with NBD-PC and NBD-GlcCer for 30 min, and lipid uptake was measured using flow cytometry. *A–C*, lipid uptake activities were plotted as the percentage of NBD-PC uptake (*A*) and NBD-GlcCer uptake (*B*) in untreated cells, with the GlcCer/PC ratio (*C*). BY4741 (WT) *erg6*Δ, *erg4*Δ, and *erg2*Δ were grown to mid-log phase and incubated with NBD-PC or NBD-GlcCer for 30 min. *D–F*, lipid uptake activities were plotted as the percentage of NBD-PC (*D*) or NBD-GlcCer (*E*) uptake for WT cells, with the GlcCer/PC ratio (*F*). Statistical variance was tested using one-way ANOVA test and Tukey's post hoc analysis. **, *p* < 0.01; ***, *p* < 0.001; ****, *p* < 0.0001. *Error bars* ± S.D. (*n* ≥ 9).

Sterol transport is linked to protein sorting through the *trans*-Golgi network and phosphatidylserine transport by the P4-ATPase Drs2 (42). Sterols and sphingolipids interact with each other, and alteration in one lipid leads to reorganization of the other in the membrane (43, 44). To test the role of ergosterol, WT cells were acutely treated with lovastatin. Lovastatin is an inhibitor of HMG-CoA reductase, a key enzyme in the mevalonate pathway, and thus blocks isoprenoid and sterol synthesis. The cells were treated with the statin for 1 h at 30 °C. We also tested plasma membrane flippase activity in the viable ergosterol biosynthesis mutants *erg6*Δ, *erg2*Δ, and *erg4*Δ by measuring NBD-PC and NBD-GlcCer transport. These perturbations reduced NBD-PC and NBD-GlcCer transport, but there was no change in the substrate specificity (Fig. 5, *A–F*). Additionally, genetic disruption of the late ergosterol synthesis pathway (45) did not alter the PM localization of Dnf1 or Dnf2 (Fig. S5, A and B). Our data suggest that genetic and pharmacologic inhibition of ergosterol production reduced the rates of lipid transport of Dnf1 and Dnf2 but did not alter their substrate preference. Sterol requirements for P-type ATPase substrate transport have been demonstrated in the Na^+^,K^+^-ATPase and Ca^2+^-ATPase (46, 47), and we anticipate that these observations may suggest a similar requirement for the completion of the P4-ATPase catalytic cycle. These data suggest that sterols may play an indirect role in supporting P4-ATPase lipid transport.

### Exogenous administration of unlabeled lipids alters Dnf2 lipid transport and substrate preference

The prior experiments indicated acute and chronic loss of endogenous sphingolipids altered the substrate specificity of Dnf2 by specifically decreasing GlcCer transport. These shifts in substrate preference were not driven by changes in intracellular signaling associated with the eisosome. We previously showed that unlabeled GlcSph (which is GlcCer without the *N*-acyl chain) and lyso-PC could inhibit NBD-GlcCer transport by Dnf2 (11). Additionally, recent cryo-EM structures of the P4-ATPase family have identified only one lipid transport pathway through these enzymes (48, 49, 50). These results suggested these two lipid substrates are competing for a common transport pathway in the P4-ATPase.

**Figure 6:**
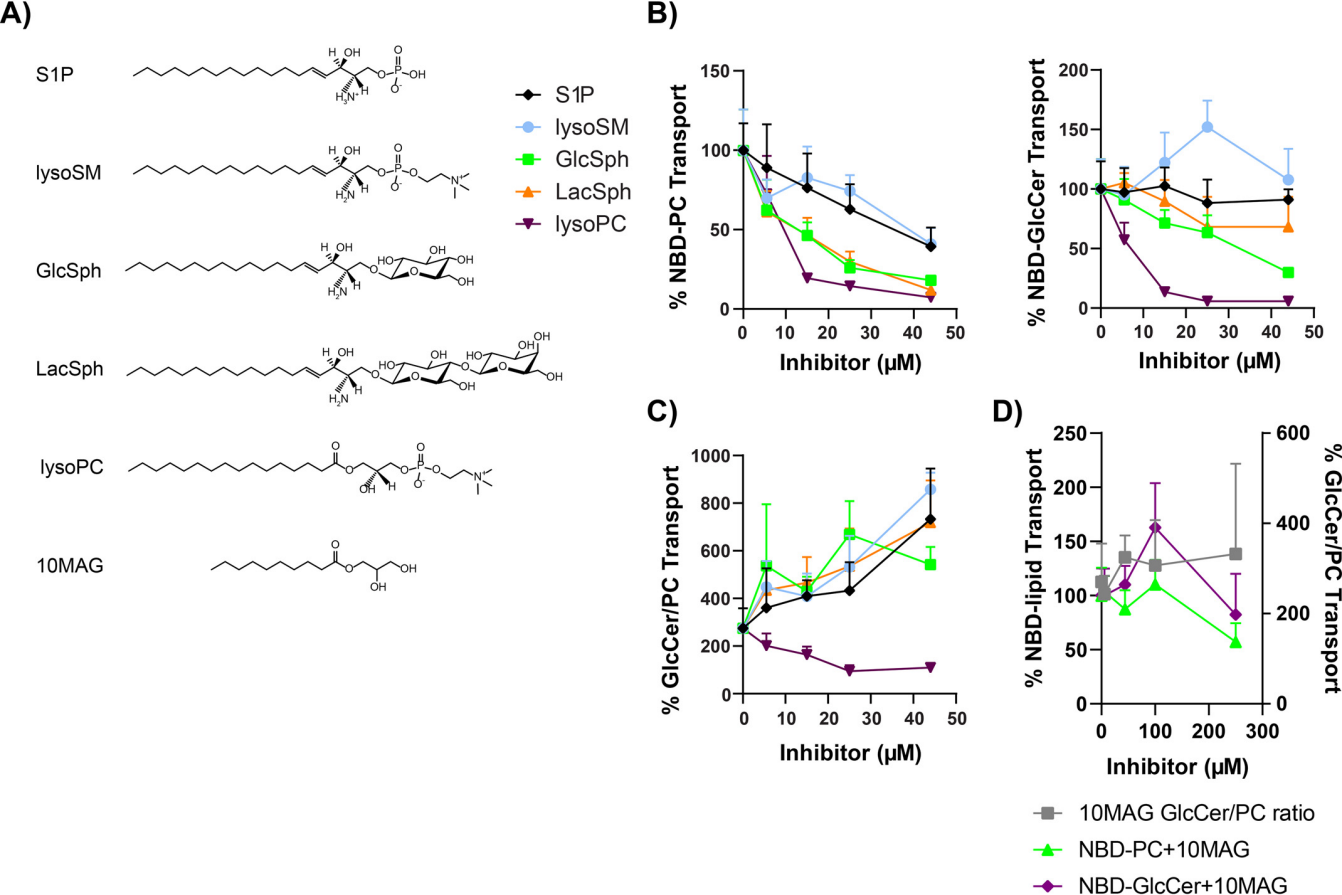
**Exogenous administration of unlabeled lipids alters Dnf2 lipid transport and substrate preference.***A*, the chemical structures of all unlabeled lipids are presented. *B*, increasing amounts of phospholipids and sphingolipids were co-administered with NBD-PC or NBD-GlcCer to *dnf1*Δ *dnf2*Δ cells with *pRS313-Dnf2* or *pRS313*. Substrate transport was plotted relative to vehicle control (0 μm). *C*, the transport data were then transformed to reflect GlcCer/PC ratios over the same concentration ranges. *D*, a lipid detergent control, 10MAG, was tested for NBD-lipid transport inhibition up to 250 μm, with NBD-lipid transport displayed on the *left y axis*, and the GlcCer/PC ratio displayed on the *right y axis*. The data presented are an XY scatter of the means ± S.D. with connecting lines (*n* ≥ 6). Note that some of NBD-GlcCer inhibition data with lyso-PC, GlcSph, and lyso-SM used in *B* were previously used for Fig. S2*E* in Ref. 11.

Here, we sought to address how acute applications of exogenous lipids to the outer leaflet of the plasma membrane influences Dnf2 activity. NBD-labeled substrates were used to measure lipid transport, and unlabeled substrate (lyso-PC and GlcSph) and nonsubstrate lipids (sphingosine-1-phosphate (S1P), lactosylsphingosine (LacSph), lysosphingomyelin (lyso-SM), and mono-acyl glycerol (10MAG)) (Fig. 6*A*) were exogenously applied to determine their influence on substrate uptake and enzyme preference (Fig. S6A). Lyso-lipids were chosen as potential inhibitors because Dnf2 appears to prefer these substrates (51). NBD-PC transport by Dnf2 was most sensitively inhibited by lyso-PC, GlcSph, and LacSph (IC_50_ = ∼9–15 μm). The inhibition by LacSph was surprising because NBD-LacCer is not a transport substrate (11). In contrast, lyso-SM and S1P inhibited NBD-PC transport weakly with an IC_50_ of ∼40 μm (Fig. 6*B*).

Unexpectedly, the pattern of NBD-GlcCer transport inhibition by these lipids was different as compared with their influence on NBD-PC transport. Lyso-PC inhibited NBD-GlcCer transport efficiently (IC_50_ = ∼8 μm), but a 4-fold higher concentration of GlcSph was required for inhibition and LacSph weakly inhibited transport over the range of concentrations tested. Lyso-SM actually stimulated NBD-GlcCer transport at low concentrations and inhibited transport at high concentrations (Fig. 6*B*; see Ref. 11 for the higher concentrations). These data were transformed as a ratio of NBD-GlcCer transport relative to NBD-PC to graphically show that these exogenous administrations of lipids altered the substrate preference of Dnf2, with most lipids inhibiting PC transport to a greater extent than GlcCer (Fig. 6*C*).

To determine whether these preferential changes in lipid transport may be due to detergent-like effects of the exogenously administered lipids, we repeated these experiments using a mono-acyl glycerol with a C10 chain in the *sn*1 position (10MAG) (Fig. 6*D*). Increasing co-administrations with 10MAG did not elicit substantial NBD-lipid transport inhibition up to 250 μm (Fig. 6*D*, *left axis*) and did not significantly change the preference of the enzyme (Fig. 6*D*, *right axis*). Co-administration of unlabeled lipid with NBD lipids may also sequester the probe in micelles away from the cells. We measured the influence of P4-ATPase expression, BSA back-extraction, and lipid co-administration on the accumulation of NBD-PC and NBD-GlcCer (Fig. S6, B and C). We found that lipid uptake in these cells is primarily driven by P4-ATPase activity, and additions of the unlabeled lipids did not significantly change the fluorescent signal of cells lacking Dnf1 and Dnf2, suggesting that the unlabeled lipids were not interfering or facilitating lipid administration or back-extraction (Fig. S6, B and C). These results support the conclusion that (i) the exogenous administration of unlabeled lipids are capable of inhibiting NBD-lipid transport, (ii) the NBD-PC and NBD-GlcCer transport pathways can be differentially effected by its lipid environment, and (iii) changing the lipid composition of the membrane can alter the observed preference of the lipid transporter.

Mammalian, plant, and fungal P4-ATPases are known to exhibit diverse substrate specificity, ranging from PS, PC, GlcCer, GalCer, PE, to SM (52). Several primary structural determinants have been mapped, suggesting how the enzyme may coordinate their amphipathic substrate(s) across the membrane (11, 33, 34, 48, 53, 54, 55, 56). These studies have yielded numerous mutant P4-ATPase variants with gain-of-function or loss-of-function substrate specificities. We predicted that if the exogenous administrations of unlabeled lipids were having a direct impact on P4-ATPase substrate recognition or transport, the inhibitory properties of these lipids would track with the known substrate specificities of these mutant P4-ATPase variants. Conversely, if the changes in membrane composition were eliciting an indirect effect on substrate transport via changes in properties such as fluidity, we would expect to see a uniform response across all P4-ATPase mutants.

A key structural feature of the P-type ATPase family is an entirely conserved Pro within the TM4 segment of the transmembrane domain. We have previously established that a conserved Gln residue located four residues N-terminal to this Pro (P–4 Gln) is a conserved determinant of GlcCer transport by human and fungal P4-ATPases. The *DNF2^Q655N^* mutation dramatically reduces NBD-GlcCer transport yet maintains substantial NBD-PC activity (11) (Fig. S7), thereby indicating that this residue plays a critical role in glycosphingolipid recognition.

**Figure 7:**
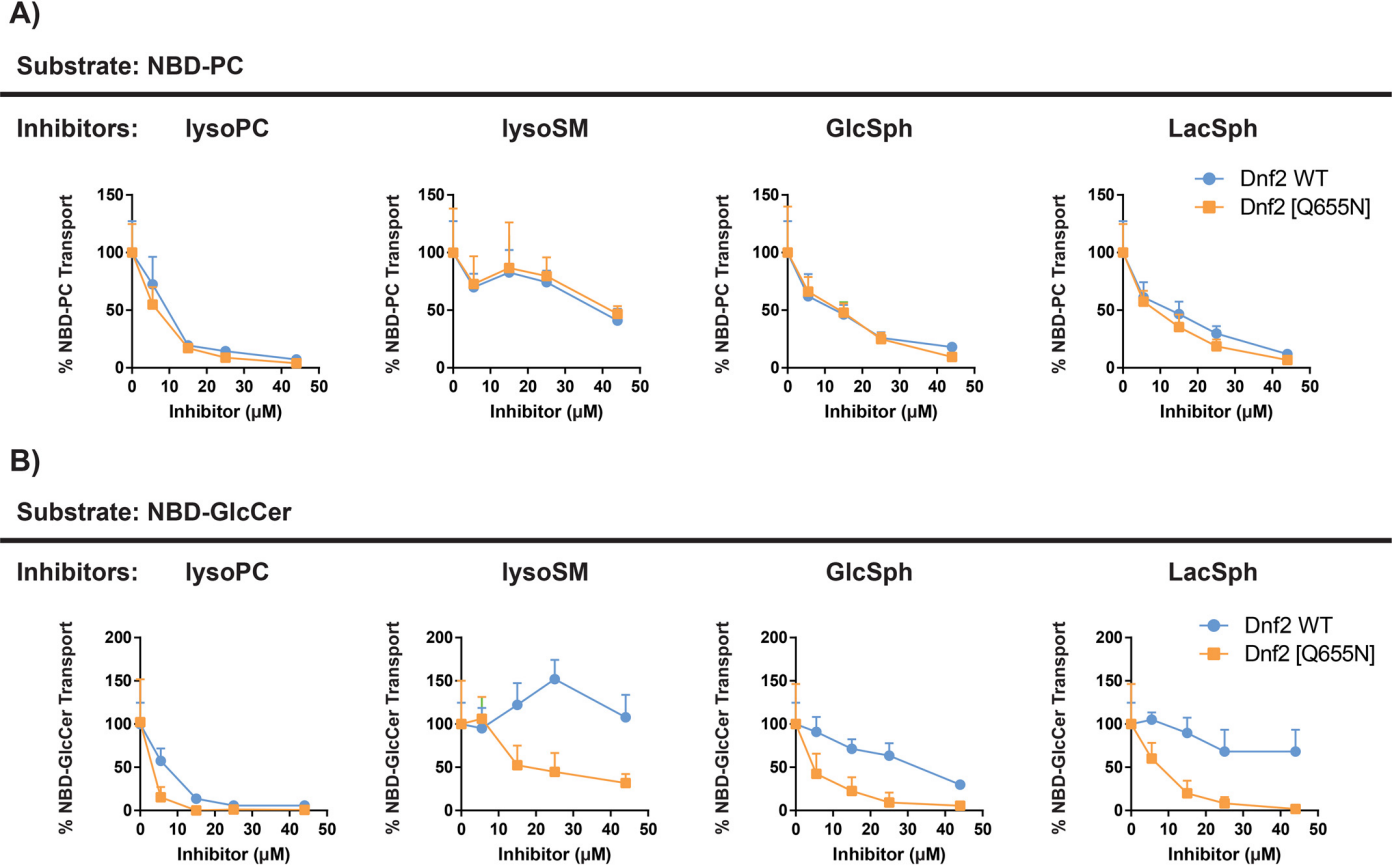
**Dnf2^Q655N^ selectively alters NBD-GlcCer inhibition profiles.** The *DNF2^WT^* and *DNF2^Q655N^* variants were tested in parallel for their ability to transport NBD-PC (*A*) and NBD-GlcCer (*B*) when co-administered with unlabeled lipids. NBD-lipid transport on the *y axis* is presented as a percentage of transport in the absence of lipid inhibitor (vehicle). The data presented are means ± S.D. (*n* ≥ 6). Note that the *DNF2 WT* NBD-GlcCer inhibition data with lyso-PC, GlcSph, and lyso-SM in *A* were previously published (11).

We expressed the *DNF2^Q655N^* variant in a *dnf1,2*Δ cell background and measured how four different unlabeled lipids altered its substrate transport profiles. We tested two classes of unlabeled lipids: substrate analogs and nonsubstrate analogs. We found that Dnf2^WT^ and Dnf2^Q655N^ enzymes responded similarly to lyso-PC, lyso-SM, GlcSph, and LacSph inhibition when translocating the NBD-PC substrate (Fig. 7*A*). However, the Dnf2^Q655N^ mutation enhanced the potency of all unlabeled lipids to inhibit NBD-GlcCer transport (Fig. 7*B*). Note that the data are normalized to the transport activity of each variant in the absence of the inhibitors. These data support the conclusion that the Q655N mutation specifically influences the ability of the Dnf2 enzyme to recognize and translocate the NBD-GlcCer substrate. Further, the enhanced potency of the unlabeled lipids for inhibiting NBD-GlcCer transport suggests that the enzyme is capable of recognizing exofacial lipid content but that translocation of NBD-GlcCer through the enzyme may be impaired.

We have previously identified an N220S TM1 substitution in Dnf1 that gains the capacity to translocate NBD-SM. This mutation was isolated from a forward genetic screen for mutations that facilitated NBD-SM transport and reduced NBD-PC uptake (53). The homologous Asn in Dnf2 is residue 258, and Dnf2^N258S^ recapitulates this NBD-SM gain-of-function while still reducing NBD-PC uptake (Fig. S7, A, B, and D). We tested whether Dnf2^N258S^ may impact NBD-PC transport or inhibition in the presence of increasing concentration of unlabeled lipids. Interestingly, NBD-PC inhibition via lyso-SM was the only transport response that was significantly altered relative to WT (Table S1 and Fig. S8). The N258S mutation reduced the potency of lyso-SM when administered with NBD-PC but not NBD-GlcCer, corroborating our previous studies of these mutants. Collectively, each of these three enzymes (Dnf2^WT^, Dnf2^N258S^, and Dnf2^Q655N^) responded differently to the changes in the exofacial membrane. By contrast, if these changes in lipid composition were eliciting an indirect influence on P4-ATPase substrate transport, these enzymes would be predicted to share their response. These unique interactions between the enzymes and their surrounding lipid environment suggest a direct relationship between the enzymology of the P4-ATPases and their surrounding glycerophospholipids and sphingolipids.

## Discussion

We report that the substrate preference of Dnf1 and Dnf2 is modulated by the composition of the membrane. Acute pharmacological inhibition of sphingolipid biosynthesis reduces GlcCer transport without altering PC transport, thus changing flippase substrate preference from glycosphingolipids to glycerophospholipids. Deletion of sphingolipid biosynthetic genes impacts transport of both substrates but again reduces GlcCer transport to a greater extent than PC or PE transport. By contrast, acute or chronic disruption of ergosterol synthesis reduced transport of GlcCer and PC equivalently. Conversely, application of exogenous sphingolipids to the outer leaflet, whether a substrate lipid or not, inhibits PC transport to a greater extent than GlcCer transport, whereas lyso-PC inhibits both PC and GlcCer transport similarly. Finally, we find that mutations that alter substrate specificity of Dnf2 also alter its response to inhibitory lipids.

*S. cerevisiae* has retained flippases for GlcCer despite losing the ability to synthesize this lipid (24), perhaps to scavenge GlcCer from decaying fungal or plant material in the environment (28, 29). We hypothesized that the loss of endogenous sphingolipids would place a greater demand on the uptake of exogenous sphingolipids, and the cells would respond by up-regulating Dnf1 and/or Dnf2 activity. This could provide an explanation for why the flippases are linked to a signaling network that also regulates sphingolipid synthesis (Fig. S3). In addition, although IPC does not appear to be a transport substrate of Dnf1/2 (10), it seemed possible that this endogenous sphingolipid (SL) could be a competitive inhibitor of GlcCer transport, in which case blocking IPC synthesis should enhance GlcCer uptake. These possibilities, however, were not supported by our observations. As measured by NBD-PC uptake, no change in Dnf1/2 activity was observed upon acute inhibition of sphingolipid synthesis. Surprisingly, however, NBD-GlcCer transport was reduced to 25–35% of that observed with mock-treated cells (Fig. 1). Thus, it appears that the substrate preference was markedly changed such that these P4-ATPases now transported PC more efficiently than GlcCer. Further, the chronic loss of mature sphingolipids induced by disruption of biosynthetic genes led to a small reduction in NBD-PC transport but a significantly larger impact on GlcCer transport and therefore elicited a similar change in substrate specificity. The specificity of this phenomenon is highlighted by our disruptions of ergosterol synthesis. Dnf1/Dnf2 transport activity is significantly reduced when ergosterol synthesis is acutely or chronically inhibited. Importantly, these changes in sterol content did not alter Dnf1/Dnf2 substrate specificity. Reducing sterol content could change the fluidity of the membrane and therefore represent an indirect influence on P4-ATPase substrate transport.

How would presence or absence of endogenous sphingolipids (SLs) influence the substrate specificity of these flippases? It is possible that the modulation of substrate preference is an allosteric effect of the membrane environment on the P4-ATPase. Lipid is initially selected through the αβ subunit interface at the exofacial leaflet; it then docks within an entry gate site formed from M1 residues and the P–4 Gln in M4 and transitions to an exit gate on the cytofacial surface where further selection of substrate can be elicited (11, 33, 34, 48, 53). Prior studies indicate that conservative amino acid substitutions within this pathway can have a major influence on flippase substrate preference. In fact, several different mutations in M1, M4, and M6 cause similar changes to Dnf2 substrate specificity as a loss of sphingolipids (11, 33, 34, 53). The acute reduction of sphingolipids in the outer leaflet may alter the conformation of Dnf2 to specifically reduce affinity for GlcCer without perturbing its ability to bind PC. Similarly, the activities of the Ca^2+^- and Na^+^/K^+^-ATPases is modulated by annular lipids that specifically associate with particular residues in this pump (57, 58). For Dnf2, a nonsubstrate lipid–induced conformational change could be a homeostatic mechanism in which cells compensate for diminished IPC-derived glycosphingolipids by reducing GlcCer transport and leaving it in the outer leaflet. Whether this reflects a loss of specific interactions between the mature sphingolipids and Dnf2 or a general response to reduced lipid density in the outer leaflet is unclear. However, the graded transport of multiple lipid substrates provided an opportunity to assess how changes in exofacial lipid concentrations impact P4-ATPase lipid transport.

Consistent with the idea that flippase substrate preference can be modulated in response to imbalances in lipid density between the two leaflets, we find that acute applications of exogenous sphingolipids, which would be expected to crowd the outer leaflet, preferentially inhibits NBD-PC transport much more than NBD-GlcCer transport (Fig. 5). Thus, adding exogenous sphingolipid to the outer leaflet has the opposite effect on substrate preference as depleting endogenous sphingolipid. For NBD-PC transport, the nonsubstrate lipids S1P and lyso-SM show a comparable dose-dependent inhibition with an IC_50_ value of ∼45 μm, whereas lyso-PC and GlcSph substrates inhibit 3–4-fold more efficiently. It is possible that S1P and lyso-SM inhibition of NBD-PC transport is primarily due to crowding of the outer leaflet, and greater potency is provided by substrates that can compete for the substrate-binding sites. In this regard, LacSph is interesting because it is not a transport substrate yet inhibits NDB-PC transport comparably to GlcSph. This can be explained if LacSph can compete for entry gate binding but is too bulky to flip from entry to exit gate.

The pattern of NBD-GlcCer inhibition by exogenous lipids was unexpected. NDB-GlcCer is transported at twice the rate of NBD-PC in WT cells (11), implying that GlcCer would have higher affinity for the flippases. However, lyso-PC is a significantly better inhibitor of NBD-GlcCer transport than is GlcSph. One possible explanation is that lyso-PC has a comparable affinity for the entry gate but does not transition from entry to exit or dissociate from the exit gate as efficiently as GlcCer. In this case, lyso-PC would be better at tying up the enzyme and reducing enzyme turnover when assayed in competition for NBD-GlcCer transport. Another oddity was the stimulation of NBD-GlcCer transport by low concentrations of lyso-SM, where NBD-PC transport was weakly inhibited (Fig. 5*B*). Similarly, this could be explained if lyso-SM, bearing the same phosphocholine headgroup as PC, could compete with endogenous lyso-PC for the entry gate but was not transported. This would reduce the number of enzymes bound unproductively to lyso-PC, perhaps holding it in the E2∼P conformational state in which NBD-GlcCer could efficiently displace lyso-SM for transport.

The involvement of the entry gate position in “sensing” membrane composition is supported by the observation that the Dnf2 Q655N mutation, which abrogates GlcCer transport much more dramatically than PC transport, also renders the remaining GlcCer transport much more sensitive to inhibition by other lipids (Fig. 7). The strong reduction in GlcCer transport seen in the *DNF2^Q655N^* variant was also characterized by a strong increase in the potency of NBD-GlcCer inhibitors. These data demonstrated that the Q655N mutations selectively impacted the coordination of NBD-GlcCer, because the inhibitory profile of NBD-PC was unchanged relative to WT. Further, if binding or translocation of the NBD-GlcCer substrate were reduced, this could enhance the potency of any inhibitors. Thus, the observed reduction in NBD-GlcCer transport *in vivo* would be caused by the concerted reduction of GlcCer affinity and the increase inhibition from the enzymes' lipid environment. Moreover, the Dnf2^N258S^ gain-of-function mutation was previously shown to increase the transport NBD-SM while reducing NBD-PC (11, 53). These effects were mirrored by changes in the potency of lyso-SM inhibition of NBD-PC transport. However, the *reduced* potency of lyso-SM as an NBD-PC inhibitor of the N258S mutant was unexpected; we had anticipated that the N258S mutant would be more susceptible to lyso-SM inhibition if this were a competitive interaction. These data led us to consider that lyso-SM may be allosterically modulating Dnf2 or altering lateral domain organization of the plasma membrane. Indeed, exogenous lyso-SM administrations did increase WT transport of NBD-GlcCer to nearly 150% that of the vehicle treatment (Fig. 7*B*). We also found that a short chain monoacylglycerol exerted no significant change in substrate specificity. These observations support the idea that we are observing specific changes in substrate preference in these studies as opposed to a more general response to membrane perturbation.

It is also possible that differential phosphorylation of Dnf1 and Dnf2 by the flippase protein kinases can modulate substrate specificity. Membrane stress induces changes in the localization of Slm1/2 proteins leading to activation of TORC2-Ypk1 signaling (59). Ypk1 directly phosphorylates Fpk1 and Fpk2 to inhibit their activity, and conversely, Fpk1/2 phosphorylates Ypk1 to inhibit its phosphorylation activity (Fig. S3) (22). Cellular status regulates the balance between Ypk and Fpk activity and therefore flippase activity. Inhibition of sphingolipid biosynthesis stimulates TORC2 and thereby up-regulates Ypk1 activity (59), which should inhibit Fpk1 and Fpk2 and cause a reduction of flippase activity (22). Indeed, we observe reduced PC and GlcCer flippase activity in mutants deficient for mature glycosphingolipids that could be caused by reduced phosphorylation of the flippases. We also see some indication that the *fpk1,2*Δ mutant displays a greater loss of GlcCer transport activity than PC transport (Fig. 1). However, in other experiments we observed a complete loss of both PC and GlcCer activity in the *fpk1,2*Δ strain (Fig. 4). The reason for these differences are unclear and may reflect subtle differences in nutrient status of the cells at the time they are assayed. We attempted to test the influence of phosphorylation by generating phosphomimetic mutations in Dnf2 (Dnf2(5S-D)) hypothesizing that this variant would be constitutively active and unresponsive to perturbation of sphingolipid synthesis. Although a phosphorylation-deficient Dnf2 mutant (5S-A) is completely devoid of flippase activity, the potential phosphomimetic Dnf2(5S-D) variant displays WT activity for both GlcCer and PC transport. However, Dnf2(5S-D) fails to display any activity in the *fpk1,2*Δ mutant. None of the single-site phosphodeficient or phosphomimetic mutants showed changes in flippase activity.^1^ These results might suggest that direct phosphorylation of Dnf2 is required for activity, but Fpk-dependent phosphorylation of other proteins is also a critical aspect of regulating flippase activity. However, the precise influence of these Ser-to-Ala and Ser-to-Asp mutations on Dnf2 structure/function are unknown, and more work is needed to understand the complex regulation of flippase activity by protein kinases.

We hypothesized that specialized membrane compartments called eisosomes may play roles in sensing changes in membrane lipid composition that would be transduced to the flippases. Inhibition of sphingolipid biosynthesis can lead to accumulation of long-chain sphingoid bases capable of stimulating the eisosome-localized Pkh1,2 proteins (60). However, deletion of a major eisosome component, Pil1, does not alter flippase transport activity or substrate specificity. Deletion of the membrane sensor Slm2 does cause a small reduction in flippase activity but no change in specificity. Interestingly, the *sur7*Δ mutant shows an increase in the GlcCer/PC ratio caused by a preferential reduction in NBD-PC transport (Fig. 3). Sur7 is a component of eisosomes and loss of this protein perturbs hydroxylation of sphingolipids by an unknown mechanism (61). The observed change in flippase substrate specificity in *sur7*Δ is similar to the effect of adding exogenous sphingolipid; perhaps *sur7*Δ causes crowding of the outer leaflet to give the similar change in flippase substrate preference.

The flippases are a unique set of transporters in that they actively modify the membrane itself rather than simply moving substrate across the membrane from one aqueous compartment to another. As such, flippases exist in a membrane environment of mixed substrate and putative inhibitors and/or allosteric modulators. Each lipid molecule has the potential to consistently sample the active site of the flippase, and their presence or absence could inherently impact the transport activity of the enzyme. We tested this hypothesis by changing the exofacial membrane composition through depletion of endogenous sphingolipids and exogenous lipid additions. We found that both substrates and nonsubstrates could influence the transport activity and preference of the flippases. Alterations to substrate transport and preference appears to be caused by the concerted influence of flippase phosphorylation, allosteric regulation, and competitive inhibition.

## Materials and methods

### Reagents

All yeast culture reagents were purchased from Sigma–Aldrich and BD Scientific. All the lipids used in this study were purchased from Avanti Polar Lipids. Myriocin was from Cayman Chemicals, whereas aureobasidin A was from Takara Bio.

### Strains and plasmid construction

Yeast strains and plasmid used in the study are listed in Tables S2 and S3. Yeast strains were grown in YPD, SD, and selective media. The yeast strains were transformed using standard transformation techniques.

Dnf2 was epitope-tagged on the C terminus with mNeonGreen by a PCR-based genomic integration method using pFA6a-mNG-Hygro as the template (62). The plasma membrane marker mCherry-Ras2 was subcloned from mCherry-Ras2-YIplac204 (63) to pRS315 (64). To create DNA constructs, PCR-amplified products were generated, and two- or three-piece Gibson assembly was performed per the manufacturer's instructions (New England Biolabs).

### Lipid uptake assays

Lipid uptake assays were conducted as described previously (11, 53). The cells were grown to the mid-log phase, and 500 μl of culture were collected for each strain. NBD-PC and NBD-GlcCer were dried and resuspended in 100% ethanol and then added to the designated ice-cold growth medium at a final concentration of 2 μg/ml. Final ethanol volumes were ≤0.5%. The cells were incubated on ice with prechilled NBD lipid + medium for 30 min. Following incubation, the cells were washed twice with prechilled SA medium (SD medium + 2% (w/v) sorbitol + 20 mm NaN_3_) supplemented with 4% (w/v) fatty acid-free BSA. Finally, the cells were resuspended in SA medium with 5 μm propidium iodide (PI), and NBD-lipid uptake was immediately measured by flow cytometry. The order in which samples were prepared and processed was varied in each experiment to reduce potential experimental error. Each experiment consisted of three independent biological replicates for each strain, repeated three times

### Inhibition assays

Inhibitor experiments were performed as defined previously (11). An illustrated depiction of the method can also be found in Fig. S1A. Briefly, the experiment was conducted as outlined above except the NBD-lipid medium was not prechilled before cellular administration. The lipids + media were allowed to remain at room temperature to improve the solubility of the inhibitors during cell application. Lipid inhibitors were solubilized in 100% ethanol and added to SD supplemented with NBD-lipid probes at the indicated final concentrations. Solubility was closely monitored, and all inhibitor lipids solubilized easily in ethanol except LacSph, which required sonication and gentle heating at 37 °C. Care was taken to ensure the final concentration of ethanol in all assay mixtures was 2.2%. No lipid insolubility was noted when the lipid mixtures were added to the media. Each experiment consisted of three independent biological replicates for each strain, repeated twice per inhibitor concentration.

### Myriocin and aureobasidin a treatment

Myriocin and aureobasidin A experiments were conducted as outlined above, except the cells were preincubated with SD + the designated concentration of each compound at room temperature for 30 min. PI staining was not enhanced with this acute treatment (Fig. S9). Each experiment consisted of three independent biological replicates for each strain, repeated three times.

### Flow cytometry

All flow cytometry experiments were conducted on a three-laser BD LSRII (BD Biosciences) operating a FACS Diva 6.1.3 software package. Single-cell populations were identified by forward- and side-scatter as illustrated in Fig. S9. PI was used to exclude dead cell populations and those with lost membrane integrity, and NBD signal was measured with FITC filters (530/30-nm band-pass filter with 525-nm long-pass filter). At least 10,000 cells were analyzed per experimental replicate.

### Fluorescence microscopy

To visualize GFP or mCherry-tagged proteins, the cells were grown to mid-logarithmic phase in YPD or minimal medium. The cells were washed with fresh media three times and resuspended in fresh SD medium. The cells were then mounted on glass slides and observed immediately at room temperature. The images were acquired using a DeltaVision Elite imaging system (GE Healthcare Life Sciences) equipped with a 100× objective lens followed by deconvolution using softWoRx software (GE Healthcare Life Sciences).

### Image analysis and quantification

Image analysis was performed as previously described (42). Overlay images were created using the merge channels function of ImageJ software (National Institutes of Health). GFP-Dnf1 and Dnf2-GFP at the plasma membrane are quantified by drawing circles inside and outside the plasma membrane using ImageJ to quantify the internal fluorescence and total fluorescence, respectively. The internal fluorescence was subtracted from the total to give the GFP intensity at the plasma membrane. At least 60 randomly chosen cells from three biological replicates (independently isolated strains with the same genotype) were used to calculate the mean and standard deviation.

### Statistical analysis

The statistical analyses were performed using GraphPad Prism 8.4.1. Variance was calculated using a one-way ANOVA test and comparisons with WT strains were tested with Tukey's post hoc analysis. The *p* values represents the significance of the data: *, *p* < 0.05; **, *p* < 0.01; ***, *p* < 0.001; and ****, *p* < 0.0001.

## Data availability

The primary data are available upon request. No material transfer agreements are required for data accessibility.

## Footnotes

1 B. K. Jain, B. P. Roland, and T. R. Graham, unpublished observations.
