# Supplementary material for: Exofacial membrane composition and lipid metabolism regulates plasma membrane P4-ATPase substrate specificity
Source: J Biol Chem. 2021 Jan 13;295(52):17997–8009. doi: 10.1074/jbc.RA120.014794 (PMC7939387; doi:10.1074/jbc.RA120.014794)
Supplement: Supplementary file 1 [file mmc1.pdf]

Supplementary Information:

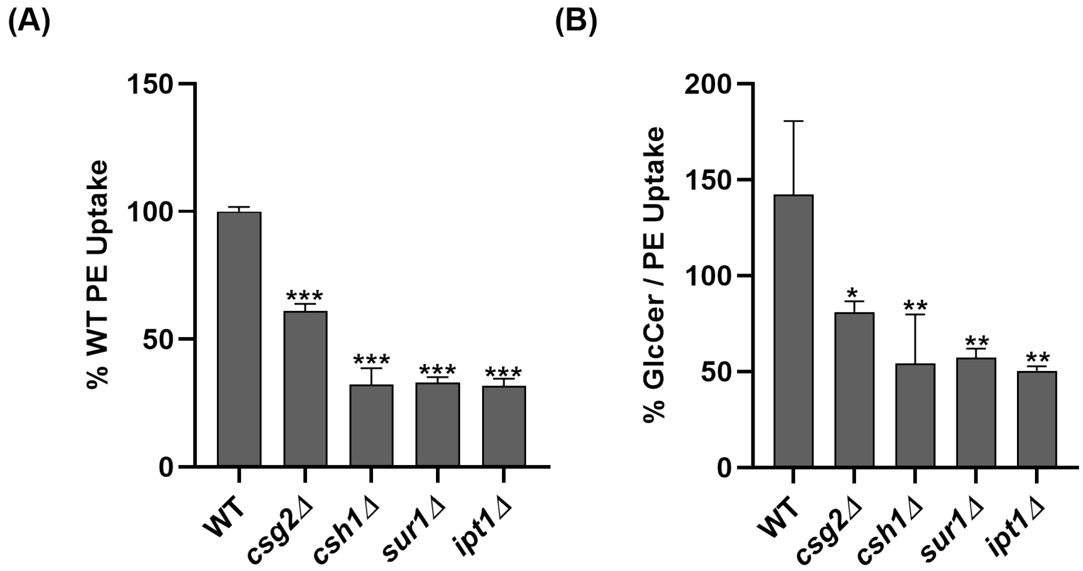

Figure S1: Sphingolipid biosynthesis mutants display reduced NBD-PE flippase activity and alters substrate preference Yeast strains BY4741 (WT), *dnf1*,2Δ, *csg2*Δ, *csh1*Δ, *sur1*Δ, and *ipt1*Δ were grown to mid-log phase and incubated with NBD-PE or NBD-GlcCer for 30 min. Lipid uptake activities were plotted as the percentage of WT NBD-PE uptake (A) with the GlcCer/PC ratio (B). Statistical variance was tested using one-way ANOVAs test and Tukey's post hoc analysis. * indicates $p < 0.05$, ** $p < 0.01$, *** $p < 0.001$. Error bars ± S.D. ($n \geq 9$).

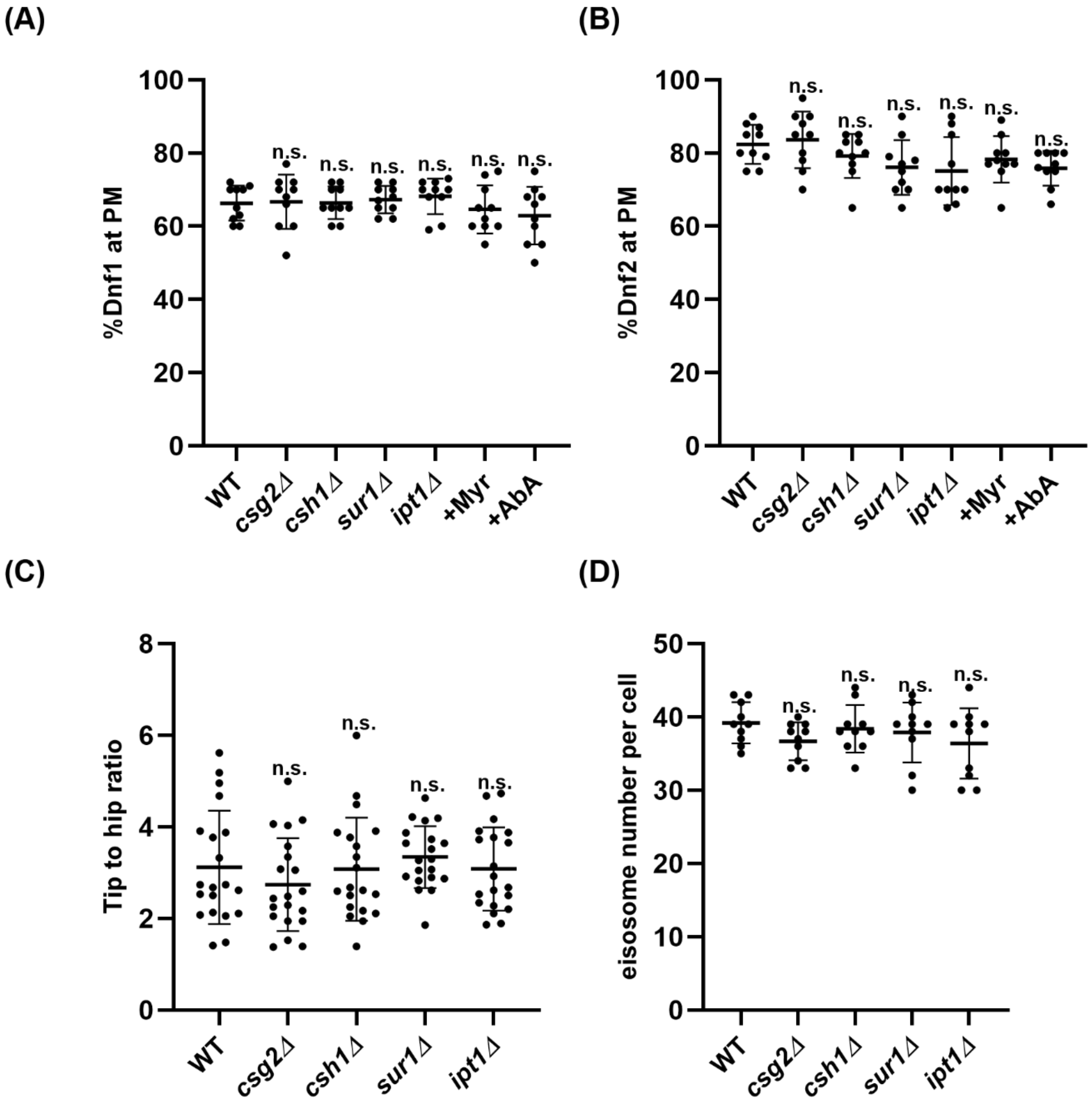

Figure S2: Localization of Dnf1 and Dnf2 to plasma membrane of sphingolipid mutants
Strains BY4741 (WT), *csg2Δ, csh1Δ, sur1Δ, ipt1Δ, and fpk1,2Δ* expressing GFP-Dnf1, Dnf2-mNG, and plasma membrane marker Ras2-mCherry were grown to mid-log phase in selective SD media. GFP-Dnf1(A) and Dnf2-GFP (B) at the plasma membrane were quantified using ImageJ by drawing circles inside and outside the plasma membrane to quantify the internal fluorescence and total fluorescence, respectively (N=60). Average is plotted as % of Dnf and Dnf2 at PM. The fluorescence at tip to hip ratio was measured by drawing a line across the bud and mother cells, then assessing the ratio of fluorescence at tip (bud) and hip (mother cells) (C) (N=20). The number of eisosomes were counted in wild type and sphingolipid mutants' strains in 30 cells. Statistical variance was tested using one-way ANOVAs test and Tukey's post hoc analysis. * indicates $p < 0.05$, ** $p < 0.01$, *** $p < 0.001$. Error bars ± S.D. n.s. indicates no significance.

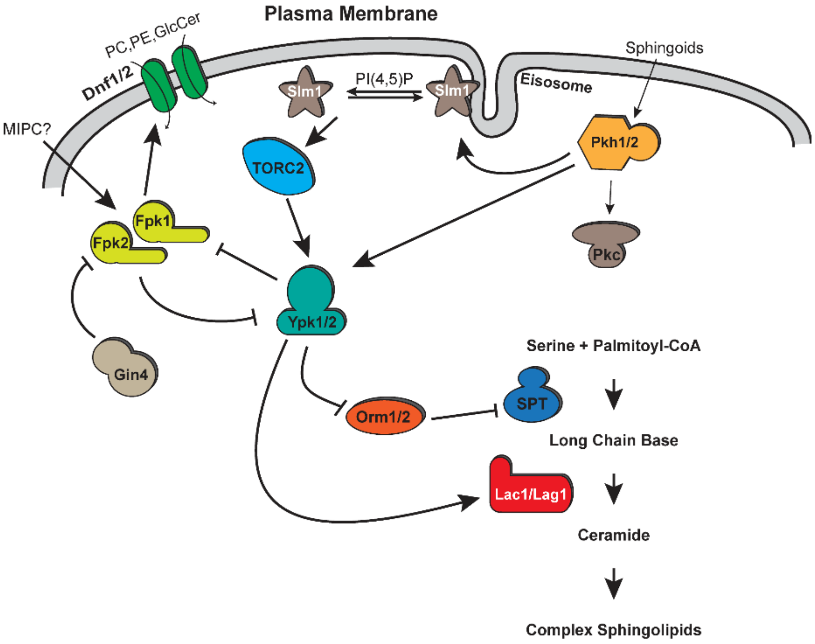

Figure S3: Fungal sphingolipid homeostatic network demonstrates the integration of cytosolic signaling with de novo sphingolipid synthesis. Inhibition or alteration in membrane composition is sensed by slm1 and slm2 proteins that activate TORC2 signaling. TORC2 activates the Ypk1,2 which modulates the activity of Fpk1,2 by balancing inhibitory/activating phosphorylation. Inhibition of Fpk1,2 leads to reduced flippase activity.

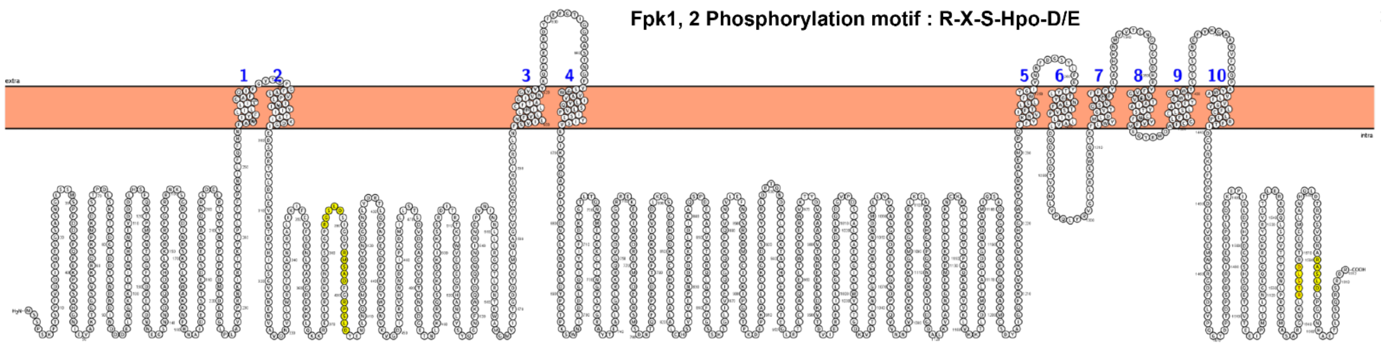

Figure S4: Topology plot of Dnf2 showing sites of phosphorylation by flippase protein kinases. Yellow marked residues represent the Fpk1,2 phosphorylation motif R-X-S- Φ -D/E in Dnf2.

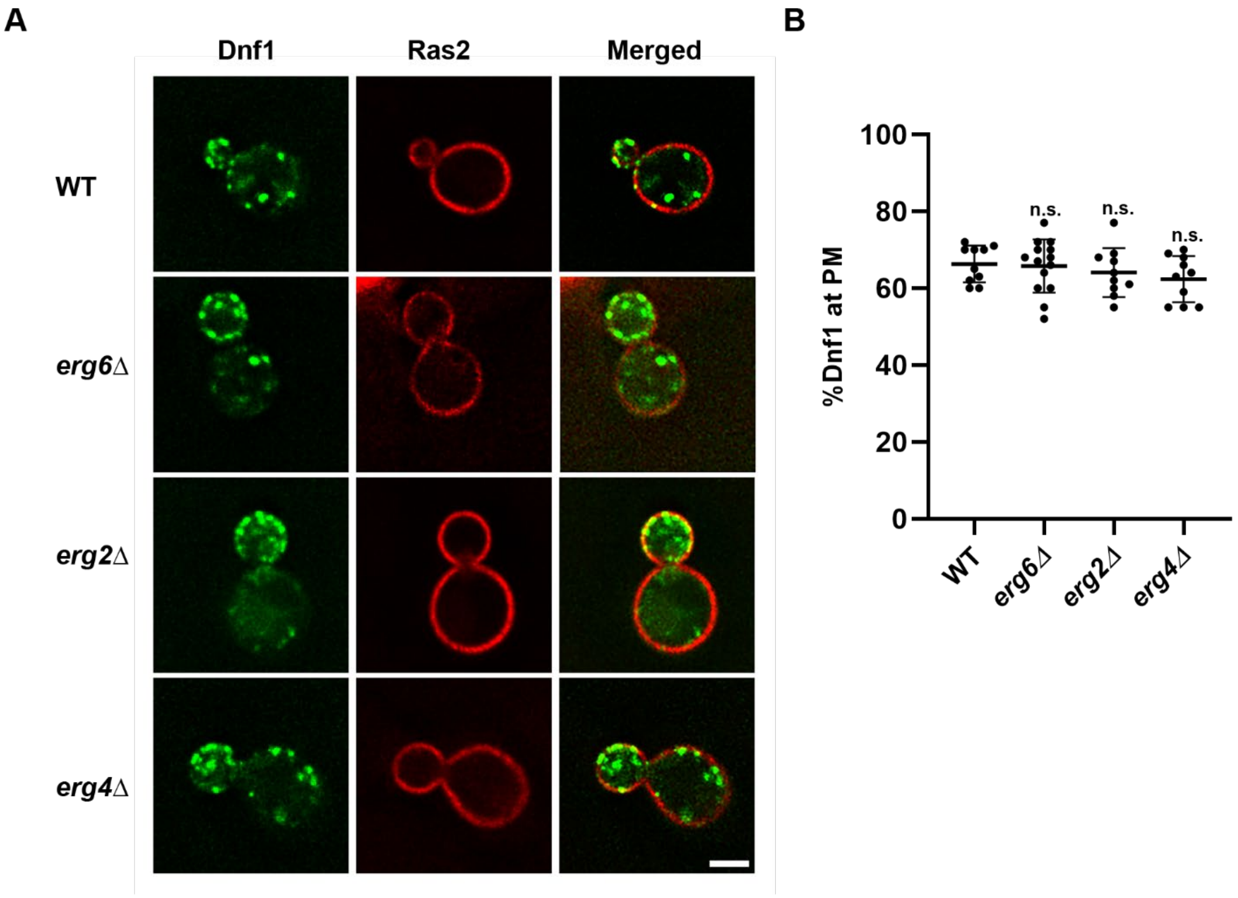

Figure S5: Localization of Dnf1 to the plasma membrane in ergosterol synthesis mutants. Wild type, *erg6*Δ, *erg2*Δ, and *erg4*Δ yeast strains expressing GFP-Dnf1 and mCheery-Ras2 were grown to mid-log phase. Cells were washed and resuspended in minimal media. Images were captured and processed using ImageJ. Representative images are shown (A) (n=60). Scale Bar = 2um. Percentage of GFP-Dnf1 at the plasma membrane are quantified as in Figure S2. Average percentage of Dnf1 at the PM is plotted ± S.D. (B) (n=40). Statistical variance was tested using one-way ANOVAs test and Tukey's post hoc analysis, n.s indicates no significance.

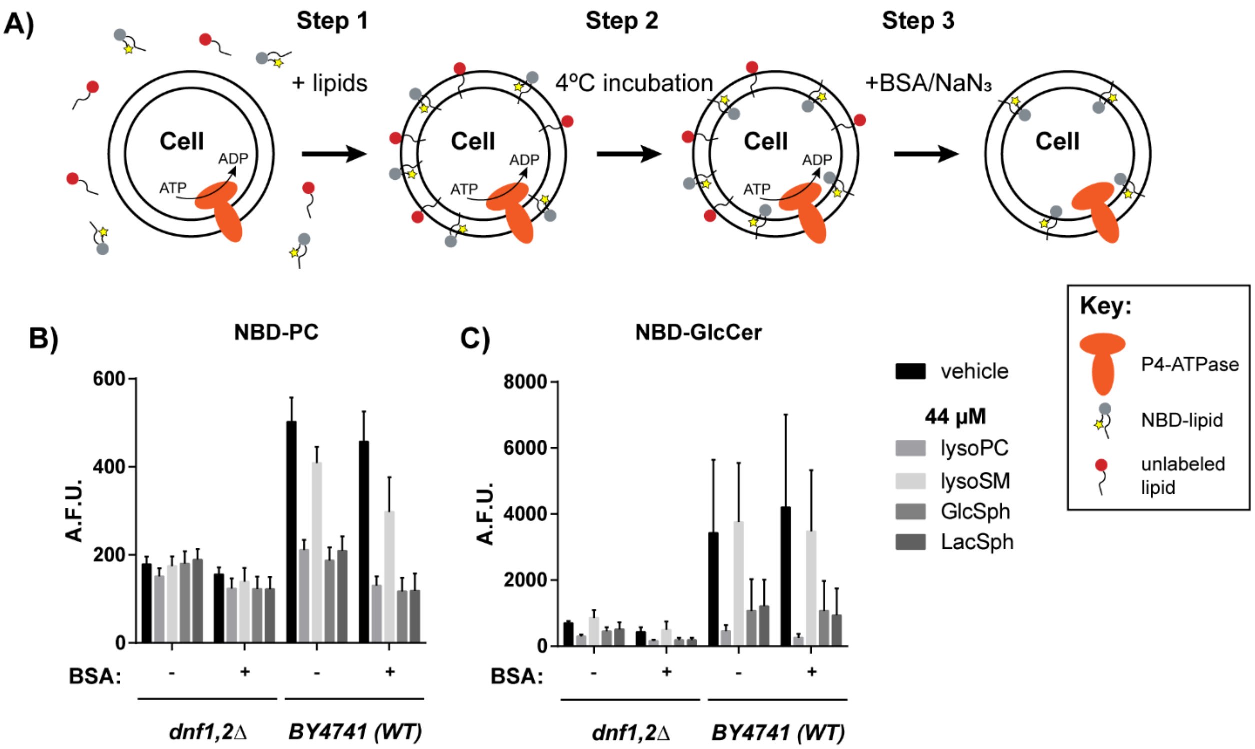

Figure S6: NBD-lipid uptake in cells is predominantly influenced by P4-ATPase active transport. An illustration of the lipid administration method (A). The co-administration assay is performed by adding assay medium lipid mixtures of NBD-lipids + unlabeled lipids to cells at room temperature for insertion, then incubation on ice at 4°C for 30 min. The cells are then washed twice with ice-cold assay medium containing 4% fatty acid-free BSA with sodium azide to back extract remaining exofacial and soluble lipids, and to inhibit P4-ATPase activity. Experiments were conducted with and without BSA in parallel to examine the relative contributions of lipid transport (*dnf1,2Δ* and *WT*), back-extraction (± BSA), and unlabeled lipid co-administration (vehicle, and 44µM lysoPS, lysoSM, GlcSph, or LacSph). NBD-PC transport was found to be largely influenced by the presence of the transporter, with and without specific unlabeled lipids (B). BSA back-extraction modestly reduced background fluorescence in *dnf1,2*Δ cells and NBD-PC uptake in WT cells (B). The co-administration of unlabeled lipids had only a minor influence on NBD-PC signal in *dnf1,2Δ* cells, suggesting the strong reduction of NBD-PC lipid uptake is due to an influence on the P4-ATPase transporters rather than a direct interaction between labeled and unlabeled lipids (B). Similar results were found when examining these same parameters with NBD-GlcCer transport (C). These experiments revealed again that the presence and activity of the P4-ATPases was the primary determinant of NBD-GlcCer uptake (C). Unlabeled lipid co-administration had measurable yet minor influences on NBD-GlcCer cell signal in *dnf1,2Δ* cells, indicating that co-administration has a modest influence on the NBD-GlcCer uptake in this assay relative to P4-ATPase expression (C). Data presented as mean ± S.D. (n≥3).

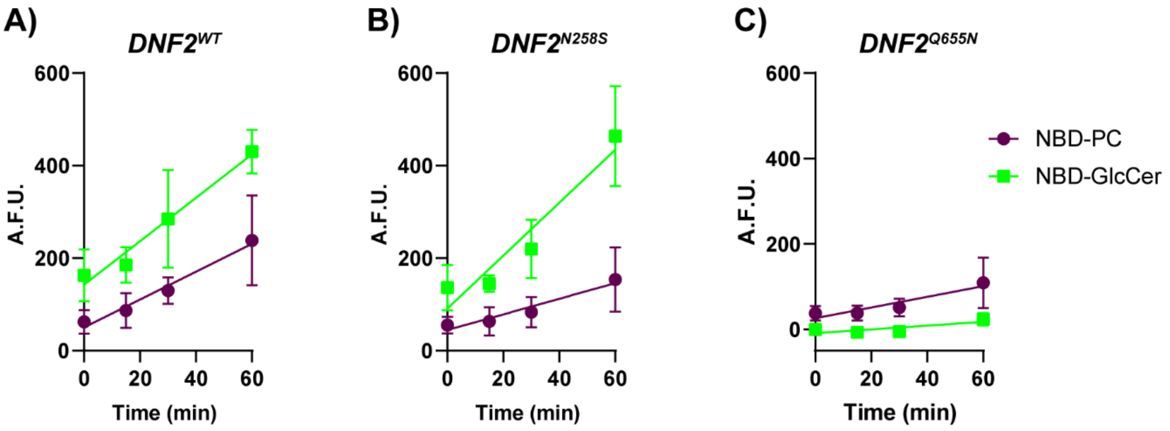

D)

AFUs/min		
	NBD-PC	NBD-GlcCer
DNF2	3.010 ± 0.497	4.685 ± 0.612
DNF2-N258S	1.690 ± 0.384	5.719 ± 0.670
DNF2-Q655N	1.249 ± 0.310	0.440 ± 0.107

Figure

Figure S7: The kinetics of NBD-PC and NBD-GlcCer transport by DNF2^{WT}, DNF2^{N258S}, and DNF2^{Q655N} enzymes expressed in *dnf1,2Δ* cells. NBD-lipids were administered to cells at 4°C and incubated for 0, 15, 30, and 60 min. Following incubation, NBD-lipids were back-extracted, and fluorescence was measured and normalized to *dnf1,2Δ* cells transformed with empty vector (*pRS313*). The kinetics of NBD-PC and NBD-GlcCer transport were assessed for *DNF2^{WT}* (A), *DNF2^{N258S}* (B), and *DNF^{Q655N}* (C). Linear regressions were fitted to the data, and the calculated slope and error are presented as arbitrary fluorescence units (AFUs) per minute (D). Data presented as mean ± S.D. (n=6).

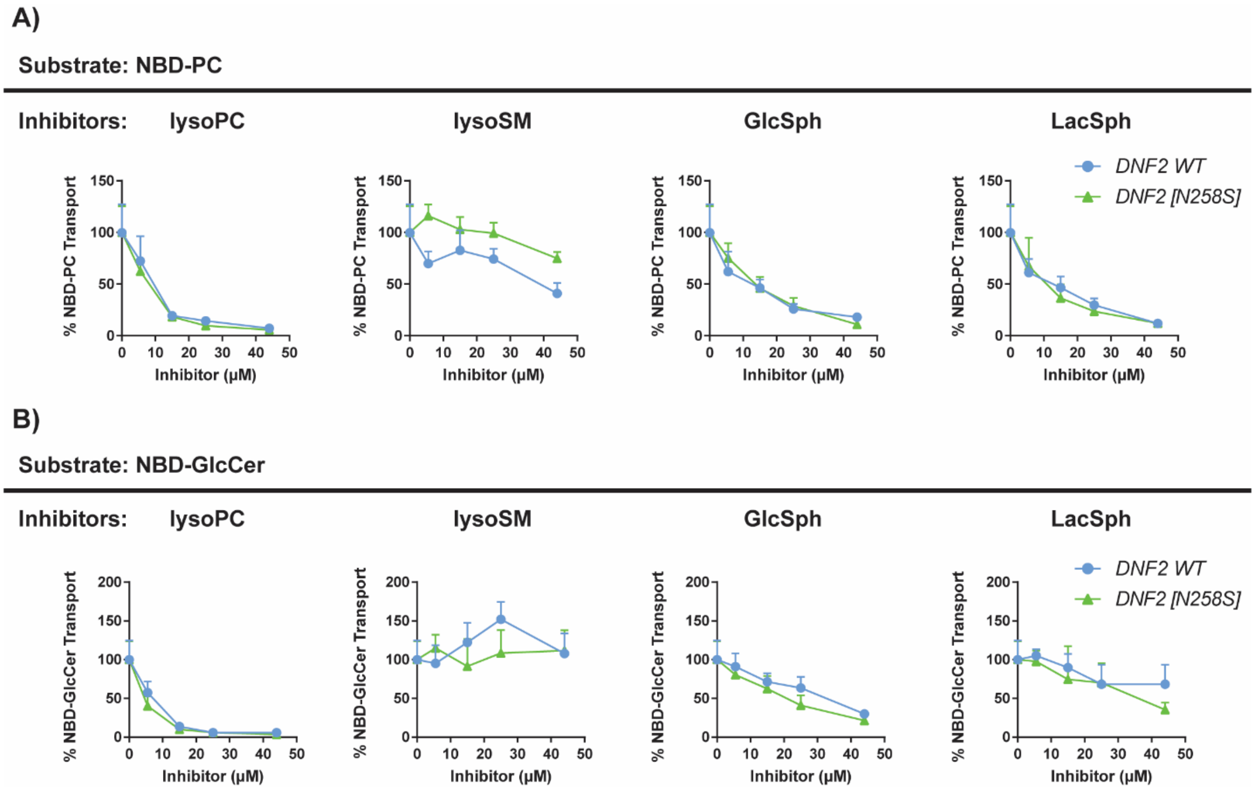

Figure S8: Dnf2^{N258S} selectively attenuates lysoSM inhibition of NBD-PC and NBD-GlcCer. The *DNF2^{WT}* and *DNF2^{N258S}* variants were tested in parallel for their ability to transport NBD-PC (A) and NBD-GlcCer (B) when co-administered with unlabeled lipids. NBD-lipid transport on the Y-axis is presented as a percentage of 0 μM lipid (vehicle). Data presented are mean ± S.D. (n≥6). Note: *DNF2 WT* NBD-GlcCer inhibition data with lysoPC, GlcSph, and lysoSM in (A) were previously published in (1).

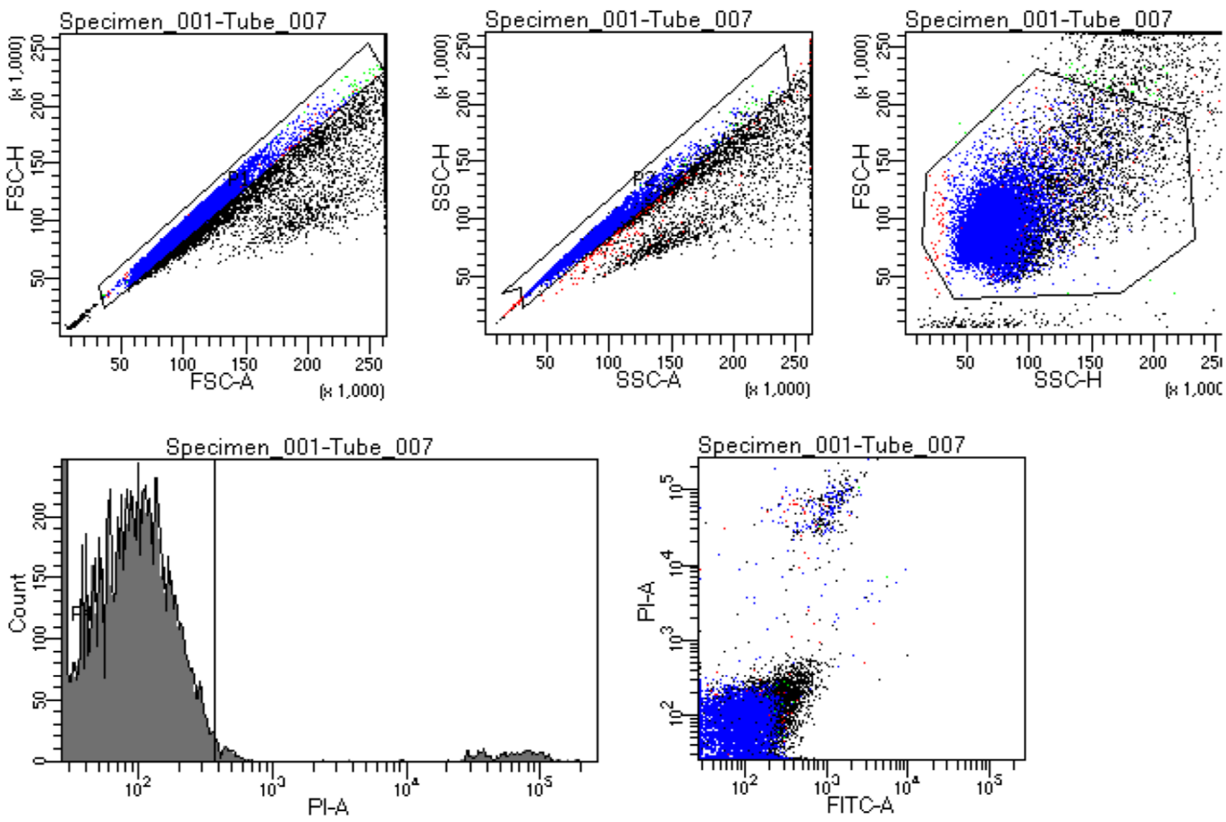

Figure S9: Gate settings used for comparative *S. cerevisiae* flow cytometry analysis. Three initial forward and side scatter gates were used to isolate single cell populations, PI staining used to select viable cells, and FITC signal was examined.

Table S2: Yeast strains used in the study

Name	Genotype	Plasmid and Source
BY4741	MATa his3Δ1 leu2Δ0 ura3Δ0 met15Δ0	Invitrogen
yBJ01	MATa his3Δ1 leu2Δ0 ura3Δ0 met15Δ0 *csg2*::KanMX	Invitrogen
yBJ02	MATa his3Δ1 leu2Δ0 ura3Δ0 met15Δ0 *csh1*::KanMX	Invitrogen
yBJ03	MATahis3Δ1 leu2Δ0 ura3Δ0 met15Δ0 *sur1*Δ::KanMX	Invitrogen
yBJ04	MATa his3Δ1 leu2Δ0 ura3Δ0 met15Δ0 *ipt1*Δ::KanMX	Invitrogen
PFY3275F	MATa *his3Δ1 leu2Δ0 ura3Δ0 met15Δ0 dnf1Δ dnf2Δ*	(5)
yBJ05	MATa *his3Δ1 leu2Δ0 ura3Δ0 met15Δ0 fpk1Δ fpk2Δ*	Present study
yBJ06	MATa his3Δ1 leu2Δ0 ura3Δ0 met15Δ0	GFP-Dnf1pRS416 mCherry-Ras2pRS425
yBJ07	MATa his3Δ1 leu2Δ0 ura3Δ0 met15Δ0 *csg2*::KanMX	GFP-Dnf1pRS416 mCherry-Ras2pRS425
yBJ08	MATa his3Δ1 leu2Δ0 ura3Δ0 met15Δ0 *csh1*::KanMX	GFP-Dnf1pRS416 mCherry-Ras2pRS425
yBJ09	MATa his3Δ1 leu2Δ0 ura3Δ0 met15Δ0 *sur1*Δ::KanMX	GFP-Dnf1pRS416 mCherry-Ras2pRS425
yBJ10	MATa his3Δ1 leu2Δ0 ura3Δ0 met15Δ0 *ipt1*::KanMX	GFP-Dnf1pRS416 mCherry-Ras2pRS425
yBJ12	MATa his3Δ1 leu2Δ0 ura3Δ0 met15Δ0 *DNF2*::mNG::Hygro	mCherry-Ras2pRS425
yBJ13	MATa his3Δ1 leu2Δ0 ura3Δ0 met15Δ0 *csg2*::KanMX *DNF2*::mNG::Hygro	mCherry-Ras2pRS425
yBJ14	MATa his3Δ1 leu2Δ0 ura3Δ0 met15Δ0 *csh1*::KanMX *DNF2*::mNG::Hygro	mCherry-Ras2pRS425
yBJ15	MATa his3Δ1 leu2Δ0 ura3Δ0 met15Δ0 *sur1*::KanMX *DNF2*::mNG::Hygro	mCherry-Ras2pRS425
yBJ16	MATa his3Δ1 leu2Δ0 ura3Δ0 met15Δ0 *ipt1*::KanMX *DNF2*::mNG::Hygro	mCherry-Ras2pRS425
yBJ18	MATa *his3Δ1 leu2Δ0 ura3Δ0 met15Δ0 dnf1Δ dnf2Δ*	pRS313(1)
yBJ19	MATa *his3Δ1 leu2Δ0 ura3Δ0 met15Δ0 dnf1Δ dnf2Δ*	pRS313-Dnf2(1)
BRY2123A	MATa *his3Δ1 leu2Δ0 ura3Δ0 met15Δ0 dnf1Δ dnf2Δ*	pRS313-Dnf2(Q655N) (1)
BRY2103A	MATa *his3Δ1 leu2Δ0 ura3Δ0 met15Δ0 dnf1Δ dnf2Δ*	pRS313-Dnf2(N258S)
yBJ20	MATa *his3Δ1 leu2Δ0 ura3Δ0 met15Δ0 dnf1Δ dnf2Δ*	pRS313-Dnf2(5S-5A)
yBJ21	MATa *his3Δ1 leu2Δ0 ura3Δ0 met15Δ0 dnf1Δ dnf2Δ*	pRS313-Dnf2(5S-5D)
yBJ368	MATa his3Δ1 leu2Δ0 ura3Δ0 met15Δ0 *erg6*::KanMX	Invitrogen
yBJ369	MATa his3Δ1 leu2Δ0 ura3Δ0 met15Δ0 *erg4*::KanMX	Invitrogen
yBJ370	MATa his3Δ1 leu2Δ0 ura3Δ0 met15Δ0 *erg2*::KanMX	Invitrogen
yBJ371	MATa his3Δ1 leu2Δ0 ura3Δ0 met15Δ0 *erg6*::KanMX	GFP-Dnf1pRS416 mCherry-Ras2pRS425
yBJ374	MATa his3Δ1 leu2Δ0 ura3Δ0 met15Δ0 *erg2*::KanMX	GFP-Dnf1pRS416 mCherry-Ras2pRS425
yBJ377	MATa his3Δ1 leu2Δ0 ura3Δ0 met15Δ0 *erg4*::KanMX	GFP-Dnf1pRS416 mCherry-Ras2pRS425

Table S3: Plasmids used in the study

pRS313	(4)
pRS313-Dnf2	(1)
pRS313-Dnf2(Q655N)	(1)
pRS313-Dnf2(N258S)	(1)
pRS313-Dnf2(5S-5A)	Present study
pRS313-Dnf2(5S-5D)	Present study
pRS416-GFP-Dnf1	(2, 3)
pFA6a-mNG-Hygro	Kathy Gould, Vanderbilt University
mCherry-Ras2-pRS425	Present study
Sur7-RFP	(6)

Supplemental References:

1. Roland, B. P., Naito, T., Best, J. T., Arnaiz-Yépez, C., Takatsu, H., Yu, R. J., Shin, H. W., and Graham, T. R. (2019) Yeast and human P4-ATPases transport glycosphingolipids using conserved structural motifs. *J. Biol. Chem.* **294**, 1794–1806
2. Baldridge, R. D., and Graham, T. R. (2012) Identification of residues defining phospholipid flippase substrate specificity of type IV P-type ATPases. *Proc. Natl. Acad. Sci. U. S. A.* 10.1073/pnas.1115725109
3. Roland, B. P., and Graham, T. R. (2016) Directed evolution of a sphingomyelin flippase reveals mechanism of substrate backbone discrimination by a P4-ATPase. *Proc. Natl. Acad. Sci. U. S. A.* 10.1073/pnas.1525730113
4. Sikorski, R. S., and Hieter, P. (1989) A system of shuttle vectors and yeast host strains designed for efficient manipulation of DNA in Saccharomyces cerevisiae. *Genetics*. **122**, 19–27
5. Hua, Z., and Graham, T. R. (2003) Requirement for neo1p in retrograde transport from the Golgi complex to the endoplasmic reticulum. *Mol. Biol. Cell*. **14**, 4971–4983
6. Spira, F., Mueller, N. S., Beck, G., von Olshausen, P., Beig, J., and Wedlich-Söldner, R. (2012) Patchwork organization of the yeast plasma membrane into numerous coexisting domains. *Nat. Cell Biol.* **14**, 640–648
